# Cystic fibrosis carriers are at increased risk for a wide range of cystic fibrosis-related conditions

**DOI:** 10.1073/pnas.1914912117

**Published:** 2019-12-27

**Authors:** Aaron C. Miller, Alejandro P. Comellas, Douglas B. Hornick, David A. Stoltz, Joseph E. Cavanaugh, Alicia K. Gerke, Michael J. Welsh, Joseph Zabner, Philip M. Polgreen

**Affiliations:** ^a^Department of Epidemiology, College of Public Health, University of Iowa, Iowa City, IA 52242;; ^b^Department of Internal Medicine, Roy J. and Lucille A. Carver College of Medicine, University of Iowa, Iowa City, IA 52242;; ^c^Department of Molecular Physiology and Biophysics, Roy J. and Lucille A. Carver College of Medicine, University of Iowa, Iowa City, IA 52242;; ^d^Pappajohn Biomedical Institute, Roy J. and Lucille A. Carver College of Medicine, University of Iowa, Iowa City, IA 52242;; ^e^Department of Biomedical Engineering, College of Engineering, University of Iowa, Iowa City, IA 52242;; ^f^Department of Biostatistics, College of Public Health, University of Iowa, Iowa City, IA 52242;; ^g^Howard Hughes Medical Institute, University of Iowa, Iowa City, IA 52242

**Keywords:** cystic fibrosis, heterozygote, CF carrier, CFTR, carrier

## Abstract

Autosomal recessive diseases, such as cystic fibrosis (CF), require inheritance of 2 mutated genes. However, some studies indicate that CF carriers are at increased risk for some conditions associated with CF. These investigations focused on single conditions and included small numbers of subjects. Our goal was to determine whether CF carriers are at increased risk for a range of CF-related conditions. Using the Truven Health MarketScan Commercial Claims database (2001–2017), we performed a population-based retrospective matched-cohort study. We identified 19,802 CF carriers and matched each carrier with 5 controls. The prevalence of 59 CF-related diagnostic conditions was evaluated in each cohort. Odds ratios for each condition were computed for CF carriers relative to controls. All 59 CF-related conditions were more prevalent among carriers compared with controls, with significantly increased risk (*P* < 0.05) for 57 conditions. Risk was increased for some conditions previously linked to CF carriers (e.g., pancreatitis, male infertility, bronchiectasis), as well as some conditions not previously reported (e.g., diabetes, constipation, cholelithiasis, short stature, failure to thrive). We compared our results with 23,557 subjects with CF, who were also matched with controls; as the relative odds of a given condition increased among subjects with CF, so did the corresponding relative odds for carriers (*P* < 0.001). Although individual-level risk remained low for most conditions, because there are more than 10 million carriers in the US, population-level morbidity attributable to the CF carrier state is likely substantial. Genetic testing may inform prevention, diagnosis, and treatment for a broad range of CF carrier-related conditions.

## Significance

Cystic fibrosis (CF) carriers are at increased risk for most of the conditions that commonly occur in people with CF. Given that there are more than 10 million CF carriers in the United States alone, the morbidity attributable to the CF carrier state is likely substantial. Thus, identifying CF carriers may aid in the prevention, diagnosis, and treatment of several common and uncommon disorders.

Cystic fibrosis (CF) is an autosomal recessive disease caused by mutations in the *cystic fibrosis transmembrane conductance regulator* (*CFTR*) gene (1–3). This gene encodes a chloride and bicarbonate channel expressed in the apical membrane of epithelial cells in multiple organ systems (1–3). People with CF have 2 defective copies of the gene and almost universally suffer from recurrent sinopulmonary infections (1–3). In addition, people with CF commonly develop endocrine, gastrointestinal, pancreatic, liver, and reproductive disorders (1).

The clinical manifestations and severity of CF vary (1, 4–6), but gene mutations producing lower levels of functional CFTR are generally associated with more severe disease (6–8). Historically, CFTR expression levels of 50% were thought to be sufficient for maintaining health (9, 10). Thus, CF carriers, having only 1 defective CFTR gene, are not considered to be at increased risk for CF-related conditions (9, 10), and CF carriers are routinely informed that they are not at increased health risk (11–13). However, some studies have found a higher-than-expected proportion of CF carriers with a limited number of CF-related conditions, including congenital bilateral absence of vas deferens (14, 15), sinusitis (16–18), pancreatitis (19–21), bronchiectasis (22–24), mycobacterial infections (25–27), and asthma (23, 28). But these investigations generally focused on single conditions, were based on small numbers of patients, did not include controls, and did not compare risk between CF carriers and people with CF. Thus, there is a need to confirm and expand on previous studies suggesting that CF carriers are at greater risk for CF-related conditions.

To establish whether CF carriers are at greater risk for CF-related conditions, we investigated the prevalence of conditions commonly associated with CF in a large population of CF carriers. We also compared the risk for each condition between CF carriers and subjects with CF. Finally, we validated our findings in an independent cohort of CF carriers identified through familial relation to subjects with CF.

## Results

### Study Data.

**Fig. 1.**
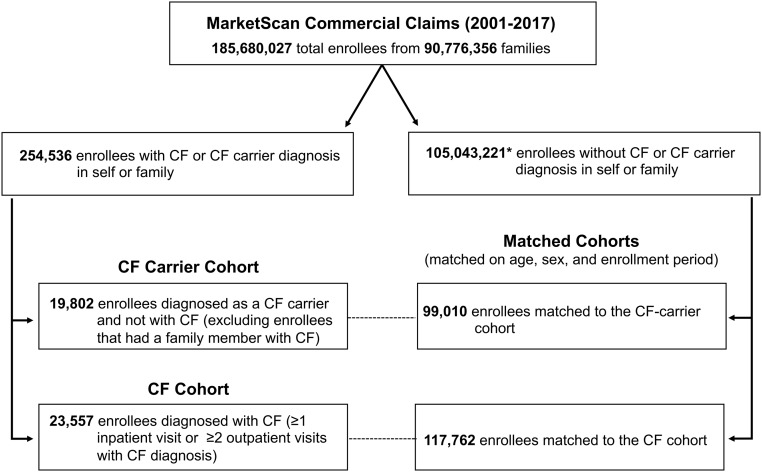
Study cohort identification and sample size. *Note that genetic screening is typically performed at birth or during the course of prenatal counseling. Thus, we were able to identify only a small proportion of enrollees who are CF carriers, and some of the subjects in the control population are very likely to be actual CF carriers.

In the CF carrier cohort, we identified 19,802 CF carriers and matched each with 5 controls, for a total of 99,010 subjects (Fig. 1). For the CF cohort, we identified 23,557 subjects with CF who were matched to a non-CF cohort of 117,762 subjects. We identified 5 matched non-CF carrier subjects for all but 6 CF cases. Table 1 summarizes baseline characteristics of the study cohorts.

**Table 1.** Baseline characteristics of the CF carrier and CF study cohorts

Characteristic	CF carriers	CF cases
Number of subjects	19,802	23,557
Female sex, *n* (%)	16,118 (81.4)	12,098 (51.4)
Age at first enrollment, *n* (%)		
0–9 y	1,662 (8.4)	6,390 (27.1)
10–17 y	718 (3.6)	4,613 (19.6)
18–26 y	4,810 (24.3)	4,818 (20.5)
27–35 y	9,850 (49.7)	3,190 (13.5)
36–45 y	2,427 (12.3)	2,175 (9.2)
≥46 y	335 (1.7)	2,371 (10.1)
Enrollment/observation time, mo		
Min–max	1–204	1–204
Mean (median)	51.3 (42)	44.0 (33)

### Prevalence of CF-Related Conditions among CF Carriers.

**Fig. 2.**
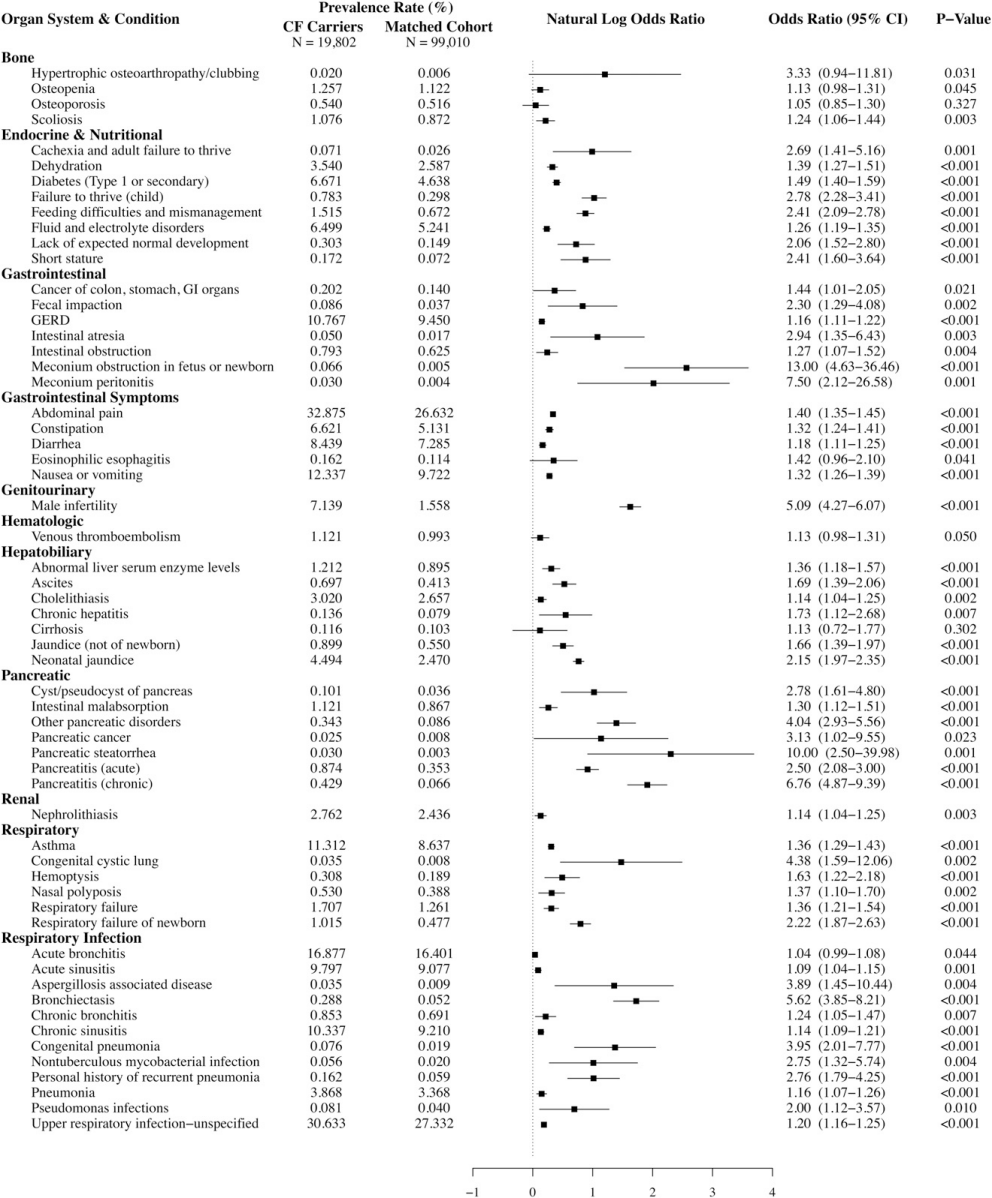
CF carriers are at greater risk for a wide range of CF-related conditions. For each CF-related health condition, the figure compares the prevalence between CF carriers and matched controls in terms of percent prevalence, OR and associated 95% CI, and *P* value for a test of the hypothesis that prevalence is greater among CF carriers. In addition, each natural log OR and its associated 95% CI are graphically depicted. The prevalence of CF-related conditions was greater in CF carriers compared with matched controls for all 59 conditions. The natural log OR for carriers was significantly greater than that for controls (*P* < 0.05) for 57 of the 59 conditions. A similar analysis between subjects with CF and matched controls is provided in *SI Appendix*, Fig. S1.

We identified 59 CF-related conditions from a literature search. All 59 conditions were more prevalent among the CF carrier cohort relative to the matched cohort. Prevalence was significantly greater (*P* < 0.05) among carriers for 57 of the 59 conditions. For each condition, Fig. 2 provides the prevalence, the odds ratio (OR) and associated 95% confidence interval (CI), and the *P* value for a 1-sided test of a null effect (i.e., an OR of 1 vs. >1). Also, natural log ORs and associated 95% CIs are graphically depicted. Carriers had significantly greater odds for conditions previously linked to the CF carrier state, such as male infertility (OR, 5.09; 95% CI, 4.27 to 6.07), chronic pancreatitis (OR, 6.76; 95% CI, 4.87 to 9.39), bronchiectasis (OR, 5.62; 95% CI, 3.85 to 8.21), and nontuberculous mycobacterial infections (OR, 2.75; 95% CI, 1.32 to 5.74). Carriers were also at greater risk for conditions not previously linked to the carrier state, such as constipation (OR, 1.32; 95% CI, 1.24 to 1.41), type I/secondary diabetes (OR, 1.49; 95% CI, 1.40 to 1.59), cholelithiasis (OR, 1.14; 95% CI, 1.04 to 1.25), failure to thrive (OR, 2.78; 95% CI, 2.28 to 3.41), short stature (OR, 2.41; 95% CI, 1.60 to 3.64), jaundice (OR, 1.66; 95% CI, 1.39 to 1.97), and scoliosis (OR, 1.24; 95% CI, 1.06 to 1.44). A similar analysis for the CF cohort is presented in *SI Appendix*, Fig. S1.

### Comparing Relative Prevalence of Conditions between CF Cases and CF Carriers.

**Fig. 3.**
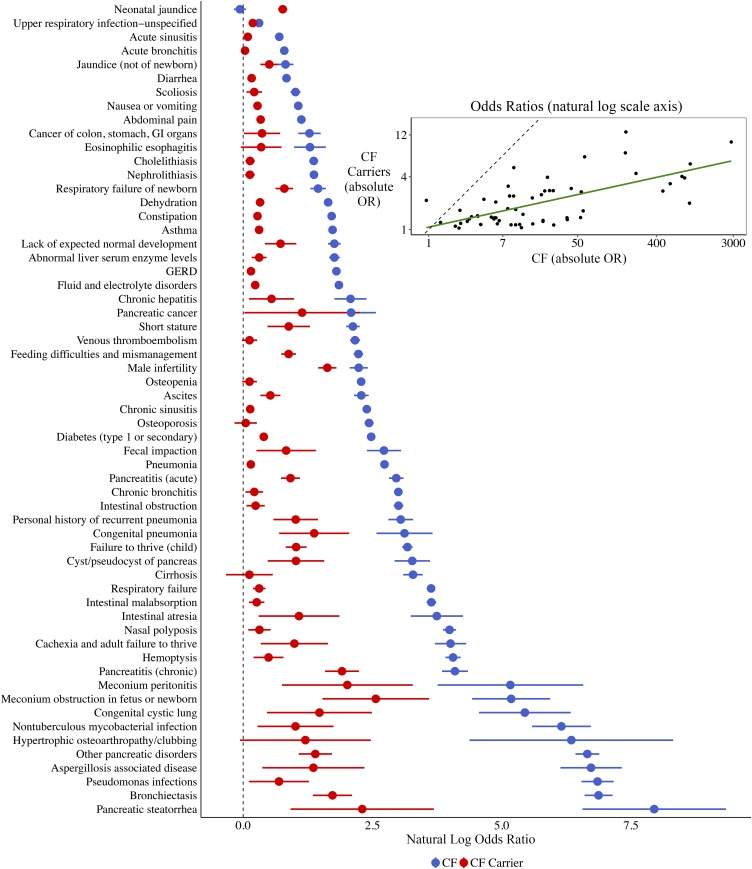
The prevalence of CF-related conditions is correlated between CF carriers and subjects with CF relative to control subjects. In the main figure, natural log ORs and corresponding 95% CIs are plotted for the various conditions for CF carriers (red) and subjects with CF (blue), ordered by the natural log OR for subjects with CF. The inset at the upper right shows a scatterplot of these values. The slope of the trend line (green) between conditions in CF carriers and subjects with CF is 0.22 (*P* < 0.001); the natural log OR of a given condition in CF carriers is ∼22% of the natural log OR in subjects with CF. The Pearson correlation between natural log ORs in the 2 cohorts across the conditions is 0.67 (*P* < 0.001).

Fig. 3 depicts the correlation in estimated natural log ORs across all conditions between CF carriers and subjects with CF, ranked by the ORs among subjects with CF. In general, as the relative odds of a given condition increase for subjects with CF, so do the corresponding relative odds for CF carriers. The Pearson correlation coefficient between the natural log ORs in the CF carrier and CF cohorts was 0.67 (*P* < 0.001).

Both CF carriers and subjects with CF were significantly more likely than controls to experience multiple CF-related conditions affecting different organ systems. Table 2 reports counts and ORs corresponding to the number of conditions or organ systems affected in both the CF carrier and CF subject cohorts relative to their respective control cohorts. For both CF carriers and subjects with CF, ORs tended to increase with the number of CF-related conditions and organ systems affected.

**Table 2.** CF carriers and subjects with CF are more likely to experience multiple CF-related conditions, and conditions that affect multiple organ systems, compared with controls

Parameter	CF carriers, *n* (%)	Controls, *n* (%)	OR (95% CI)	CF, *n* (%)	Controls, *n* (%)	OR (95% CI)
Conditions						
0	5,819 (29.39)	38,286 (38.67)	0.61 (0.59–0.63)	1,734 (7.36)	55,564 (47.18)	0.06 (0.06–0.06)
1–2	7,895 (39.87)	36,007 (36.37)	1.16 (1.13–1.2)	4,634 (19.67)	39,644 (33.66)	0.48 (0.46–0.49)
3–4	3,589 (18.12)	15,246 (15.40)	1.23 (1.18–1.28)	4,361 (18.51)	14,268 (12.12)	1.69 (1.63–1.75)
5–6	1,529 (7.72)	6,057 (6.12)	1.30 (1.22–1.38)	3,668 (15.57)	5,316 (4.51)	4.17 (3.98–4.37)
7–8	603 (3.05)	2,206 (2.23)	1.39 (1.27–1.53)	2,845 (12.08)	1,921 (1.63)	8.91 (8.37–9.48)
9–10	222 (1.12)	824 (0.83)	1.36 (1.17–1.58)	2,077 (8.82)	668 (0.57)	18.12 (16.53–19.87)
>10	145 (0.73)	384 (0.39)	1.91 (1.57–2.31)	4,238 (17.99)	381 (0.32)	89.1 (78.97–100.54)
Organ systems						
0	5,819 (29.39)	38,286 (38.67)	0.61 (0.59–0.63)	1,734 (7.36)	55,564 (47.18)	0.06 (0.06–0.06)
1	5,853 (29.56)	27,644 (27.92)	1.08 (1.05–1.12)	3,081 (13.08)	31,140 (26.44)	0.41 (0.4–0.43)
2	4,111 (20.76)	17,604 (17.78)	1.22 (1.17–1.27)	4,005 (17.00)	17,449 (14.82)	1.18 (1.14–1.23)
3	2,311 (11.67)	9,258 (9.35)	1.30 (1.23–1.36)	4,004 (17.00)	8,156 (6.93)	2.87 (2.76–3)
4	1,076 (5.43)	4,037 (4.08)	1.37 (1.27–1.47)	3,560 (15.11)	3,580 (3.04)	6.16 (5.85–6.48)
>4	632 (3.19)	2,181 (2.20)	1.48 (1.35–1.63)	7,173 (30.45)	1,873 (1.59)	38.42 (36.03–40.96)

ORs corresponding to the presence of a certain number of distinct conditions and the presence of a certain number of total organ systems affected are provided between CF carriers and controls and between subjects with CF and controls. Conditional logistic regression models were used to estimate the odds of a subject experiencing a given number of conditions/organ systems affected. For both CF carriers and subjects with CF, the relative odds of experiencing a given number of conditions or organ systems affected increased with the number of conditions/systems.

### Prevalence of CF-Related Conditions in the Familial Validation Cohort.

We found little evidence of selection bias. People who undergo genetic testing may differ in substantial ways from those who do not, and selection bias may occur because we identified CF carriers via genetic screening. Thus, we built a familial validation cohort of CF carriers composed of mothers of CF patients (before the CF child’s birth). *SI Appendix*, Fig. S2 presents prevalences and ORs across conditions in the familial validation CF carrier cohort. In general, results in the validation cohort tended to mirror those in the primary cohort. A total of 42 conditions pertained to adult women and had sufficient prevalence for analysis; of these, 40 conditions had higher prevalence among CF carriers, and 28 were statistically significant. OR point estimates for most conditions in our validation cohort were very similar to the results obtained in our primary analysis. However, due to the limited size of the validation cohort (only 2,185 carriers), CIs tended to be wider, and some conditions lacked sufficient data for analysis. Of the 28 significant conditions, 26 had estimated ORs contained in or greater than the CIs reported in the primary cohort. CIs for 40 of the 42 conditions overlapped between the primary and validation cohorts; intervals for the other 2 conditions (i.e., asthma and dehydration) were higher in the validation cohort.

### Sensitivity Analyses for Results in Primary CF Carrier Cohort.

Our findings cannot be explained by misclassification bias, that is, people with CF misclassified as CF carriers due to undetectable *CFTR* mutations on 1 allele. We estimate that given a population of 19,802 CF carriers identified through genetic screening, we would expect approximately 58 to 117 subjects to be misclassified (*SI Appendix*, Methods S2). However, for the majority of the conditions that we studied, to obtain similar results, more than 3,055 CF carriers would need to represent misclassified cases of CF (*SI Appendix*, Table S3). Moreover, when considering results across all the conditions reportedly associated with CF, we were unable to reproduce results similar to ours, even after 2 million simulated trials (*SI Appendix*, Table S4). In addition, false discovery could not explain our results. We obtained significance for 57 out of 59 conditions, and our simulation analysis suggested that false discovery could explain these findings for less than 1 condition, specifically 0.43 conditions on average (*SI Appendix*, Table S5). Finally, we found little evidence of ascertainment bias. When we excluded CF carriers with evidence that genetic screening was performed due to suspicion of CF, our results remained consistent, and estimates changed only slightly (*SI Appendix*, Table S6).

## Discussion

Our results suggest that CF carriers are at increased risk of developing a wide range of CF-related conditions across multiple organ systems. This risk was observed in a cohort of CF carriers identified by genetic testing and in a secondary cohort of mothers of children with CF, before the child with CF was born. CF carriers exhibited a similar, albeit muted, phenotype compared with individuals with CF. Specifically, the relative log ORs across the 59 conditions analyzed were correlated between CF carriers and subjects with CF. Thus, as the relative odds of a given condition increased among subjects with CF, so did the corresponding relative odds for carriers. In addition, CF carriers were more likely than controls to suffer from multiple CF-related conditions. Taken together, our results call into question the idea that CFTR expression levels associated with the CF carrier state are sufficient to completely protect subjects from CF-related conditions.

Previous investigators have reported that CF carriers are at increased risk for some the conditions we reported, including infertility (14, 15), pancreatitis (19–21), and sinopulmonary infections (16–18, 25–27, 29, 30). However, for the majority of conditions investigated, an increased risk has not been associated with the CF carrier state. For example, we found evidence of an elevated risk for type I diabetes or secondary diabetes (conditions associated with insulin insufficiency), dehydration, electrolyte disorders, constipation, newborn respiratory failure, and short stature. In fact, for 57 of the 59 conditions evaluated, we found that CF carriers were at significantly increased risk compared with controls (*P* < 0.05).

Despite the large relative ORs we report for some of these conditions, it is important to stress the difference between relative and absolute risk. Fig. 2 shows not only the ORs for CF-related conditions, but also the prevalence of each condition in our study population. For conditions such as chronic pancreatitis, the relative risk is very high for carriers (OR, 6.76; 95% CI, 4.87 to 9.39), but the absolute risk of pancreatitis is low, even for CF carriers (0.429 per 100) in our sample. Thus, the vast majority of CF carriers will never develop pancreatitis. Accordingly, the difference between relative and absolute risk should be considered when interpreting our results.

What is the physiological basis for the increased risk of CF-related conditions in carriers? Carriers have ∼50% as much CFTR anion channel activity as controls (31, 32), and in some epithelia, anion transport is known to be reduced in carriers. For example, carriers have β-adrenergic agonist-stimulated sweat secretory rates that are intermediate between values for controls and people with CF (33, 34); sweat secretory rates depend on CFTR-mediated Cl^−^ secretion by epithelia in the sweat gland secretory coil. Another example is that carriers have pilocarpine-stimulated sweat Cl^−^ concentrations that lie between control and CF values (35); sweat Cl^−^ concentration depends on CFTR-mediated Cl^−^ absorption by epithelia in the sweat gland duct. This abnormality may explain the increased risk for dehydration and fluid and electrolyte disorders found in this study. In airway epithelia, Cl^−^ secretion by carriers does not differ from that of controls (32); however, a rate-limiting step for Cl^−^ secretion by airway epithelia is Cl^−^ entry across the basolateral membrane and not exit through apical CFTR (32, 36–38). In contrast, apical CFTR limits the rate of HCO_3_^−^ secretion across airway epithelia, and CF carriers have approximately one-half as much HCO_3_^−^ secretion as controls (32). Decreased HCO_3_^−^ secretion and an abnormally acidic airway surface liquid may contribute to lung disease in people with CF (3, 39–41) and may explain the elevated risk of lung disease that we found for carriers. These observations suggest that additional studies of epithelial Cl^−^ and HCO_3_^−^ secretion, especially under stimulated conditions, and elucidation of the rate-limiting steps for transepithelial anion transport may yield greater understanding of how reduced CFTR function may predispose or not to disease in various organs.

An increasing number of CF carriers are being identified. Knowing that carriers are at greater risk for CF-related conditions may help clinicians and patients choose more effective screening and prevention approaches. Being a CF carrier may provide motivation for avoiding other disease-risk factors, such as excessive alcohol use (e.g., pancreatitis), and may help inform screening or preventive-treatment strategies (e.g., gastrointestinal cancer). In addition, identifying patients with these conditions who are also CF carriers may someday have treatment implications. Newer drugs developed to treat CF by correcting defects in CFTR function may augment Cl^−^ and HCO_3_^−^ transport (1). For example, the CFTR potentiator ivacaftor increases the open-state probability of wild-type CFTR (42, 43), and its effect on the nonmutant allele might be sufficient to attenuate or prevent the phenotype seen in CF carriers. Although the high cost of these medications may preclude their use, they may have a role in conditions with limited treatment options (e.g., multidrug-resistant nontuberculous mycobacterial infections).

A primary limitation of our study is that we were able to identify only a small proportion of CF carriers in the study population. Most CF carriers that we identified were likely screened during reproductive care or through newborn screening. However, only a minority of parents receive genetic screening, and newborn screening for CF is intended to detect newborns with CF and only occasionally detects CF carriers. Thus, some control subjects were likely CF carriers. This misclassification biases our findings toward the null hypothesis, however. Moreover, the carriers who we identified through evidence of genetic screening were relatively young, limiting our ability to investigate the risk for conditions more prevalent among older populations (e.g., osteoporosis, cirrhosis). Thus, our risk estimates should not be interpreted precisely, especially for conditions that affect predominantly older populations.

Our study has some additional limitations. First, our data do not allow the identification of specific CFTR mutations, and it is possible that our results are not generalizable to all CF carriers. However, most CF carriers (∼70% in the US) have the F508del mutation (44, 45). Second, our data also do not allow for direct analysis of demographic factors (e.g., race). Third, because all of our subjects had health insurance, our results might not be generalizable to other populations. Fourth, we were dependent on diagnostic codes with varying sensitivity and specificity to identify CF-related conditions. Thus, CF-related conditions could not be validated through chart review, and the prevalence of CF-related conditions in the general population might not have been precisely captured. Finally, our primary analysis was performed using a cohort of CF carriers identified through genetic screening, and it is possible that individuals who underwent genetic screening may differ from those not screened (i.e., selection bias). However, our validation cohort did not rely on genetic screening, and the similar findings we obtained using the validation cohort provides further evidence that selection bias does not explain our results.

In conclusion, our results suggest that CF carriers are at increased risk for most conditions associated with CF. Given that there are more than 10 million CF carriers in the US, the disease burden attributable to the CF carrier state is likely substantial. We believe that identifying CF carriers may aid the prevention, diagnosis, and treatment of several common and uncommon disorders. The widespread availability of CF genotyping made this discovery possible, and with increased genetic screening, it may be possible to apply a similar approach to people who are carriers of other recessive genetic diseases.

## Materials and Methods

### Study Data.

We conducted a population-based retrospective matched-cohort study using the Truven Health MarketScan Research Database from 2001 to 2017. This database includes longitudinal deidentified inpatient, emergency department, and outpatient records, along with pharmaceutical claims for beneficiaries, family members, and dependents.

### Identification of Study Cohorts.

To estimate the health risks attributable to the CF carrier state, we constructed a CF carrier cohort and a matched non-CF carrier cohort. For comparison, we constructed a cohort of subjects with CF and a similarly matched control cohort using the following selection criteria (*SI Appendix*, Table S1 reports the diagnostic codes).

#### CF carrier cohort.

First, subjects were diagnosed as a “CF gene mutation carrier” through genetic testing. Second, they were never diagnosed with CF. Third, subjects were excluded if a family member was diagnosed with CF, because members of these families may use more healthcare.

#### CF cohort.

Subjects with CF were identified based on a CF diagnosis recorded during at least 1 inpatient stay or during 2 or more outpatient visits (46).

#### Non-CF carrier matched-control cohorts.

Each subject in both the CF carrier cohort and CF cohort was matched on sex, age during the observation period, and total months of enrollment to 5 subjects without a diagnosis of CF or CF carrier status. To minimize inclusion of unrecognized CF carriers, we excluded control subjects from families with CF cases or CF carriers.

### Selection of CF-Related Conditions.

We performed a literature review to identify conditions commonly attributable to CF and selected 59 conditions for evaluation (*SI Appendix*, Methods S1). *SI Appendix*, Table S2 lists the 59 CF-related conditions that we considered and their diagnosis codes.

### Statistical Analysis.

#### Prevalence of CF-related conditions among CF carriers.

Our primary hypothesis was that CF carriers are at increased risk for conditions associated with CF compared with matched control subjects. We first computed the prevalence of each CF-related condition per 1,000 enrollees in a cohort. We then estimated the odds of subjects in the CF carrier cohort being diagnosed with a given condition relative to subjects in their matched cohort using a conditional logistic regression model. Prevalence rates and ORs were computed for each condition based on a diagnosis during any inpatient or outpatient visit.

#### Comparison between CF carriers and CF cases.

Our secondary hypothesis was that CF carriers exhibit a similar, but substantially muted, phenotype compared with subjects with CF. We explored this hypothesis in 2 ways. First, we repeated our primary analysis in the CF cohort, then compared ORs for each condition between CF carriers and subjects with CF to determine whether the greater relative prevalence among subjects with CF corresponded to a greater prevalence among CF carriers. For the purpose of this comparison (and for graphical depiction), we used (natural) log-transformed ORs, which aligns with the logistic regression scale. We used the Pearson correlation coefficient and linear regression to estimate the association between respective natural log ORs in the CF and CF carrier cohorts across all considered conditions.

Second, we compared the likelihood of experiencing multiple conditions. For each cohort, we computed both the total number of CF-related conditions and number of organ systems involved for each individual across study cohorts. Next, for a specific number of total conditions or organ systems (e.g., 0, 1 to 2, 3 to 4), we used conditional logistic regression to estimate the odds of subjects in the CF carrier or CF cohort having that number of conditions/organ systems affected relative to their respective control cohort. We compared trends across organ systems between the CF carrier and CF cohorts.

#### Prevalence of CF-related conditions among CF carriers in a familial validation cohort.

Carriers in our primary cohort were identified because they were genotyped; such individuals might differ from other enrollees, potentially leading to selection bias. We could not determine the exact reason for genetic screening using administrative data, and screening might have been performed based on clinical suspicion of CF (i.e., ascertainment bias). Carriers also might behave differently once a carrier status is known. Alternatively, individuals with more frequent healthcare interactions may be more likely to be screened. Therefore, to account for any selection biases associated with how we identified CF carriers via genetic screening, we built an independent validation cohort of CF carriers selected based on familial relation and not on genetic screening codes. Specifically, we identified all mothers without CF who had a child diagnosed with CF, where the child’s birth could be captured during the mother’s observation period to ensure maternity. In addition, we restricted our analysis to the period before the diagnosis of CF in the child, to remove any potential biases associated with health-seeking behavior after a child was diagnosed with CF. Finally, we repeated our primary analysis using this validation cohort. Because some conditions do not apply to adult women (e.g., male infertility, congenital pneumonia), we excluded some conditions from our analysis (*SI Appendix*, Methods S4).

#### Sensitivity analyses.

We conducted 3 sensitivity analyses. First, to determine whether misclassification bias (i.e., undetected cases of CF due to rare *CFTR* mutations) could explain our findings, we used a bootstrapping approach to simulate the effect of including misclassified subjects with CF in the cohort of CF carriers (*SI Appendix*, Methods S2). We estimated the degree of misclassification (i.e., cases of CF misclassified as CF carriers) required to produce our results and compared this with expected misclassification rates for standard genetic screening panels. Second, to account for the possibility of significant findings due to multiple comparisons, we estimated the number of results that we obtained across all 59 conditions that could be expected due to false discovery. Because of the complex and interdependent relationship between our selected conditions and organ systems, we used a simulation analysis to compute an empirical false discovery rate (*SI Appendix*, Methods S3). Third, because genetic screening may occur when CF is suspected, ascertainment bias might have impacted our primary carrier cohort. Thus, we eliminated CF carriers identified by genetic screening performed in response to clinical suspicion of CF (e.g., screening in response to bronchiectasis). We then repeated our primary analysis after excluding any enrollees who had either sweat chloride test results or a procedure code indicating reimbursement for an expanded or nonstandard screening panel.

#### Materials and data availability.

The Truven Health MarketScan Commercial Claims and Encounters (CCAE) and Medicare Supplemental (MDCR) research databases are widely used and are publicly available for purchase. This database is proprietarily owned and may be purchased from IBM Watson Health. The data use agreements stipulate that researchers at the University of Iowa are legally not permitted to release these data to researchers outside of the University of Iowa; however, identical data can be obtained from IBM Watson Health.

All of the methods and algorithms required to reproduce our results are described herein or in the *SI Appendix*. All of the International Classification of Diseases 9/10 codes used to identify study participants and diseases of interest are provided in the *SI Appendix*. In addition, the pseudocode and programming scripts to reproduce our results are available on the public repository GitHub (https://github.com/aarmiller/cf-carrier).

## Supplementary Material

Supplementary File
