# Supplementary material for: Cystic fibrosis carriers are at increased risk for a wide range of cystic fibrosis-related conditions
Source: Proc Natl Acad Sci U S A. 2019 Dec 27;117(3):1621–7. doi: 10.1073/pnas.1914912117 (PMC6983448; doi:10.1073/pnas.1914912117)
Supplement: Supplementary File [file pnas.1914912117.sapp.pdf]

Cystic-fibrosis carriers are at risk for cystic fibrosis-related conditions

SUPPLEMENTARY APPENDIX

Table of Contents

Methods S1: Methods used to review and select conditions associated with cystic fibrosis……..2

Methods S2: Methods used to simulate the effect of cases of CF misclassified as CF carriers …3

Methods S3: Methods used to estimate empirical false discovery rate from multiple comparisons……………………………………………………………………………………6

Methods S4: Methods used to identify and analyze validation cohort …………………………..7

Table S1: Diagnosis codes used to identify study cohorts………………………………………..8

Table S2: Diagnosis codes used to identify conditions associated with cystic fibrosis.………….9

Table S3: Results of potential misclassification analysis for individual conditions…….………16

Table S4: Results of potential misclassification across all conditions …………………………19

Table S5: Results of multiple comparison analysis for empirical false discovery rate ………...20

Table S6: Results of sensitivity analysis for association bias associated with screening ………21

Figure S1: Prevalence and odds ratios for each condition analyzed for CF patients and matched cohort …..24

Figure S2: Prevalence and odds ratios for each CF-related condition in the independent validation cohort……………………………………………………………………………25

References:.……………………………………..……………………...………..…….26

www.pnas.org/cgi/doi/10.1073/pnas.1914912117

Methods S1: Methods used to review and select conditions associated with cystic fibrosis.

To identify cystic fibrosis-related conditions, we performed a literature search designed to find literature reviews describing conditions and symptoms related to CF. The following search was used to identify a total of 1,364 review papers in PubMed.

> Search (cystic fibrosis[Title] AND symptoms) OR (cystic fibrosis[Title] AND conditions) OR (cystic fibrosis[Title] AND cancer) Filters: Review; Publication date from 2000/01/01 to 2018/12/31; Humans; English.

From this list, all abstracts were reviewed. We excluded papers focused on treatment, screening, management or diagnosis of CF and refined this list to include a total of 122 papers to review in detail. [1-122] Our goal was to investigate conditions related to CFTR mutations. Thus, we excluded some conditions for the following reasons. First, we excluded conditions indirectly related to CF. For example, we excluded urinary incontinence and rectal prolapse as they are most likely related to coughing and constipation, respectively, and not directly related to CFTR mutations. We excluded pulmonary hypertension for the same reason. Similarly, we excluded depression and related conditions as they are more common among patients with chronic illnesses in general. Second, we excluded conditions that we considered to be a result of CF-related care or exposure to healthcare: *Clostridium difficile* colitis, acute and chronic kidney injury, fibrosing colonopathy, and the MRSA-carrier state. Third, we excluded conditions without robust ICD-9-CM/ICD-10-CM codes (e.g., *Burkholderia cepacia* complex, small intestine bacterial overgrowth). Finally, we excluded female infertility from our condition list. The risk was reported to be elevated among CF patients, and we found increased risk among CF carriers, however, we think that women with infertility may be more likely to have CF screening, and thus be diagnosed as a CF-carrier as part of their prenatal care.

Methods S2: Methods used to simulate the effect of cases of CF misclassified as CF carriers.

We performed a simulation analysis to determine if the results observed in our study could be explained by cases of CF that were misclassified as CF carriers due to rare CFTR mutations not detectable by standard screening panels. The objective of our simulation analysis was to test the following (null and alternative) hypotheses:

> H_0: *There is no difference between CF carriers and matched control patients (without genetic mutations), and the observed effects for a given condition can be attributed to misclassified patients with CF.*
>
> H_A: *CF carriers are at increased risk for a given condition, and the observed effect is greater than what would be expected simply from misclassified cases of CF.*

To test this hypothesis, we created a simulated CF-carrier cohort comprised as (1) patients not identified as CF carriers or with CF, and (2) patients with CF; these two cases represented CF carriers (with no observable effect) and misclassified patients with CF (with an effect), respectively. For each simulation, we randomly drew a simulated cohort based on a hypothesized misclassification rate. Specifically, for a given misclassification rate α we selected $19{,}802 * \alpha$ patients with CF and $19{,}802 * (1 - \alpha)$ patients without evidence of CFTR mutations. A summary of the simulation algorithm can be found below.

We performed three basic simulation analyses. **First**, we used a bootstrapping approach to compute *p*-values corresponding to a misclassification rate α that would be expected based on population prevalence of CF and CFTR mutations, and screening accuracy for standard CFTR panels. A description of how expected misclassification rates were calculated can be found below. For each condition, we performed 100,000 simulation trials and computed the number of trials that produced odds ratios greater than those reported in Figure 2. Results of this simulation analysis can be found in Table S3.

Second, we estimated the average misclassification rate and number of misclassified cases that would be necessary to generate the results reported in our study. For each condition, we performed a grid search over various misclassification values to identify the misclassification rate that, on average, produced odds ratios equal to those reported in Figure 2. Specifically, we varied α between 0 and 1, in 0.001 increments. For each value we ran 2,000 simulated trials and computed the average estimated odds ratio. We then used these values to estimate the misclassification rate needed to generate our results. Results of this simulation analysis can also be found in Table S3.

Third, we performed a simulation to test if the results we obtained across all conditions could be explained by misclassification. Specifically, we estimated the total number of conditions, out of the 59 evaluated, that we would expect to generate similar estimates given expected misclassification rates. For each simulation trial, we estimated the odds ratio across all of the 59 conditions evaluated. We then computed the total number of conditions for which the estimated odds ratios were greater than or equal to the values reported in Figure 2. We performed one

million trials of this final simulation, for both the upper and lower bound on the expected misclassification rates (described below). Results of this simulation are summarized in Table S4.

Simulation Algorithm. The following steps summarize the algorithm that was used for each of the simulations described above. For a given condition, misclassification rate α, and number of trials *n*, the following was performed:

1. Take a parameter $\alpha \in [0,1]$ representing the fraction of CF carrier cases we expect to be misclassified (i.e., these are actually cases of CF) and create a simulated CF carrier cohort, containing misclassified cases of CF and CF carriers that are identical to control patients.
    a. *Misclassified CF cases*: Randomly replace a fraction α of the CF carriers with known CF cases, matched based on age, sex and enrollment time.
    b. *Simulated CF carriers with no effect*: Randomly replace the remaining fraction $(1-\alpha)$ of the CF carriers with control cases (without CF or CF-carrier markers), drawn randomly from the cohort of patients not included in the study population and matched on age, sex and enrollment time.

2. Compute the odds ratio for a given condition between the original control cohort and the simulated CF carrier cohort.

3. Repeat, *n*-times per value of α.

4. Compute the percentage of times that the simulated CF carrier cohort produced an odds ratio "as extreme" as the odds ratio obtained in our original cohort (p-value) or return the average estimated odds ratio (and corresponding percentiles) across all trials.

Note on drawing matched CF cases: In step 1a) some of the age-sex-enrollment strata contained fewer CF cases than CF carriers, and some contained no CF cases. For older CF carriers (e.g., age > 40), it may be harder to find exact age-sex-enrollment matches because of the decreased life expectancy associated with CF. Thus, for any strata where fewer CF cases could be identified than the number of CF carriers, we implemented the following strategy to identify CF cases that were as closely matched as possible. For a given strata, we performed the following:
1. If the number of CF cases was greater than or equal to the number of CF carriers in a given strata, we drew CF cases without replacement for step 1A from the above algorithm.
2. If the number of CF cases was less than the number of CF carriers and greater than 0 in a given strata, we drew CF cases with replacement if all CF cases were drawn. For example, if a given strata contained 15 CF carriers and only 8 CF cases, and during a single simulation trial 9 CF carriers were selected to be replaced with CF cases, we then drew the 9th CF case with replacement.
3. If the number of CF cases was 0 in a given strata, we looked for matches in the following order:
    a. First, we relaxed the constraint that CF cases have a CF diagnosis in at least 2 outpatient visits and instead looked for exact age/sex/enrollment matches among all enrollees that were diagnosed with CF during at least one inpatient or

outpatient visit. If at least one exact match could be found, we proceeded according to the steps 1 or 2, as outlined above. If no exact matches could be found, we proceeded to the following step.

b. We looked for CF cases, among enrollees with any CF diagnosis that were closest in age but still had the same sex and enrollment time. This final criterion allowed us to identify matches for all enrollees using an average age threshold of 1.6 years.

Simulation Parameters. We assumed that CF screening panels typically capture 80-90% of genetic mutations.[123,124] Thus, a CF carrier would be correctly identified with probability 80-90%. Similarly, a CF case would be correctly identified with probability 64-81% (e.g., 0.8^2 to 0.9^2) but would be labeled as a CF carrier with probability 18-32% (i.e., 2*0.9*0.1 to 2*0.8*0.2). We also assumed that the likelihood of being a CF carrier was approximately 1/37 while the probability of CF was 1/2500.[125] Thus, using Bayes Theorem we would expect approximately 0.295-0.590% of our observed CF carrier cohort, or approximately 58 to 117 enrollees, to be misclassified patients with CF. Note: although these values depend on the demographic information not contained in our enrollment data (e.g., race and ethnicity), previous estimates of carrier risk following negative test results have been reported between 1/380 (0.263%) for Ashkenazi Jewish populations and 1/170 (0.588%) for African American populations.[123] Thus, the parameter values that we use to bound our simulation analysis, namely 0.295-0.590%, entirely encapsulate the range of expected misclassification rates for individuals of different races or ethnicities.

The calculated rate of expected misclassification can be summarized by the following formulas:

$$P(CF|1\ CFTR\ Mutation\ Detected) = \frac{P(1\ CFTR\ Mutation\ Detected\ |CF) * P(CF)}{P(1\ CFTR\ Mutation\ Detected)}$$

where,

$$P(1\ CFTR\ Mutation\ Detected) = P(CF) *\ P(1\ CFTR\ Mutation\ Detected\ |CF) + P(CF\ Carrier) *\ P(1\ CFTR\ Mutation\ Detected\ |CF\ Carrier)$$

Thus, for a screening mutation detection rate of 80% we would expect a misclassification rate of:

$$= \frac{(1/2{,}500) * 0.32}{(1/2{,}500) * 0.32 + \left(\frac{1}{37}\right) * 0.80} \approx 0.00588516$$

And for a screening detection rate of 90% we would expect a misclassification rate of:

$$= \frac{(1/2{,}500) * 0.18}{(1/2{,}500) * 0.18 + \left(\frac{1}{37}\right) * 0.90} \approx 0.002951264$$

Methods S3: Methods used to estimate empirical false discovery rate from multiple comparison.

Our primary analysis involves the testing of multiple hypotheses across various CF-related conditions; thus, we performed a sensitivity analysis for the number of estimates that might be attributable to false discovery. Because many of the conditions and organ systems that were analyzed are inter-related, we used a simulation analysis to estimate an empirical rate of false discovery. Similar to the analysis described in Methods S2, we performed analysis by building multiple simulated cohorts of non-carriers then repeating our study analysis. For a given simulation trial, we first replaced each carrier with a randomly drawn non-carrier with the same sex, enrollment period and age. We then re-drew the matched control cohort, using the same criteria described in the study. Finally, we repeated our primary prevalence analysis and computed the total number of conditions that had p-values less than, or equal to, those reported in Table 2. We performed 10,000 trials for this simulation, and results are summarized in Table S5.

Methods S4: Methods used to identify and analyze validation carrier cohort.

We first identified all children with a diagnosis of CF using the diagnosis codes reported in Table S1. We used the first child diagnosed with CF as the index diagnosis, in households with multiple children with CF. We also eliminated families where either parent was diagnosed with CF. Next, we identified mothers (female enrollees listed as either the primary beneficiary or spouse) within the child's family. To better ensure genetic maternity, we eliminated all mothers whose observation period did not overlap with the child's birth. Finally, we identified the point where the child, or any child, was first diagnosed with CF and then truncated the mother's observation period to the time from her first enrollment to the point where her first child with CF was diagnosed.

Selection of matched controls. In order to identify matched controls in the most consistent manner possible, we first matched all CF carriers (i.e., mothers) to controls based on sex, months of total enrollment and age over the entire study period. However, because we restricted analysis to the period before the child was diagnosed with CF, we truncated the observation period for both CF carriers and matched controls to the same time span prior to the CF diagnosis. For example, suppose a carrier mother was followed for 24 months, but the child's first CF diagnosis occurred 9 months after the start of the enrollment period. We started by matching this carrier to 5 controls that could be followed for 24 months. We then truncated the observation period for both the carrier and matched controls to the first 9 months of enrollment. We opted to perform matching on the full enrollment period followed by truncation, rather than matching on the truncated period in order to control for possible differences in individuals followed for different lengths of time. For example, enrollees with shorter enrollment windows may differ in meaningful ways from enrollees with longer enrollment periods.

Conditions excluded from analysis: A number of the conditions we analyzed are not applicable to adults or women. Thus, we excluded a number of conditions from analysis in our validation cohort. These exclusions were confirmed by our panel of CF experts who selected the original list of conditions for analysis. Male infertility was excluded due to enrollee sex. Meconium peritonitis, meconium obstruction, neonatal jaundice, congenital cystic lung, newborn respiratory failure, congenital pneumonia, and childhood failure to thrive, were excluded as these conditions apply only to newborns or children.

Table S1: Diagnosis codes used to identify study cohorts.

The following International Classification of Disease (ICD) diagnosis codes were used to identify CF carriers and subjects with CF. These codes were also used to identify households with potential prevalence of *CFTR* mutations. Note: X indicates that all nested sub-codes were used.

Diagnosis Description	ICD-9-CM Codes	ICD-10-CM Codes
Cystic Fibrosis Carrier	V83.81	Z14.1
Cystic Fibrosis	277.0X	E84.X

Table S2: Diagnosis codes used to identify conditions associated with cystic fibrosis.

Note: The HCUP Clinical Classification Software (CCS) categories were used to identify codes for some conditions. [126,127] For simplicity, some of the ICD-10 codes have been replaced with the CCS category code that was used. A complete list of the individual ICD-10 codes can be found at: https://www.hcup-us.ahrq.gov/toolssoftware/ccs/ccs.jsp.[126,127]

Condition Group	Condition	ICD-9-CM Codes	ICD-10-CM Codes
Bone	Hypertrophic osteoarthropathy /clubbing	7312, 7815	M8940, R683
Bone	Osteopenia	73390	M8580
Bone	Osteoporosis	7330	M810, M816, M818
Bone	Scoliosis	73730	M4120
Endocrine & Nutritional	Cachexia and adult failure to thrive	7837, 7994	R64, R627
Endocrine & Nutritional	Diabetes (Type 1 or Secondary)	249, 25001, 25003, 25011, 25013, 25021, 25023, 25031, 25033, 25041, 25043, 25051, 25053, 25061, 25063, 25071, 25073, 25081, 25083, 25091, 25093, 7902, 7915, 7916, V4585, V5391, V6546	E109, E1010, E1011, E1021, E1022, E1029, E10311, E10319, E10321, E103211, E103212, E103213, E103219, E10329, E103291, E103292, E103293, E103299, E10331, E103311, E103312, E103313, E103319, E10339, E103391, E103392, E103393, E103399, E10341, E103411, E103412, E103413, E103419, E10349, E103491, E103492, E103493, E103499, E10351, E103511, E103512, E103513, E103519, E103521, E103522, E103523, E103529, E103531, E103532, E103533, E103539, E103541, E103542, E103543, E103549, E103551, E103552, E103553, E103559, E10359, E103591, E103592, E103593, E103599, E1036, E1037X1, E1037X2, E1037X3, E1037X9, E1039, E1040, E1041, E1042, E1043, E1044, E1049, E1051, E1052, E1059, E10610, E10618, E10620, E10621, E10622, E10628, E10630, E10638, E10641, E10649, E1065, E1069, E108, Z794, E089,

			E099, E139, R7301, R7302, R7303, R7309, R739, R81, R824, Z4681, Z9641, E0800, E0801, E0810, E0811, E0821, E0822, E0829, E08311, E08319, E08321, E083211, E083212, E083213, E083219, E08329, E083291, E083292, E083293, E083299, E08331, E083311, E083312, E083313, E083319, E08339, E083391, E083392, E083393, E083399, E08341, E083411, E083412, E083413, E083419, E08349, E083491, E083492, E083493, E083499, E08351, E083511, E083512, E083513, E083519, E083521, E083522, E083523, E083529, E083531, E083532, E083533, E083539, E083541, E083542, E083543, E083549, E083551, E083552, E083553, E083559, E08359, E083591, E083592, E083593, E083599, E0836, E0837X1, E0837X2, E0837X3, E0837X9, E0839, E0840, E0841, E0842, E0843, E0844, E0849, E0851, E0852, E0859, E08610, E08618, E08620, E08621, E08622, E08628, E08630, E08638, E08641, E08649, E0865, E0869, E088, E0900, E0901, E0910, E0911, E0921, E0922, E0929, E09311, E09319, E09321, E093211, E093212, E093213, E093219, E09329, E093291, E093292, E093293, E093299, E09331, E093311, E093312, E093313, E093319, E09339, E093391, E093392, E093393, E093399, E09341, E093411, E093412, E093413, E093419, E09349, E093491, E093492, E093493, E093499, E09351, E093511, E093512, E093513, E093519, E093521, E093522, E093523, E093529, E093531,

			E093532, E093533, E093539, E093541, E093542, E093543, E093549, E093551, E093552, E093553, E093559, E09359, E093591, E093592, E093593, E093599, E0936, E0937X1, E0937X2, E0937X3, E0937X9, E0939, E0940, E0941, E0942, E0943, E0944, E0949, E0951, E0952, E0959, E09610, E09618, E09620, E09621, E09622, E09628, E09630, E09638, E09641, E09649, E0965, E0969, E098, E1300, E1301, E1310, E1311, E1321, E1322, E1329, E13311, E13319, E13321, E133211, E133212, E133213, E133219, E13329, E133291, E133292, E133293, E133299, E13331, E133311, E133312, E133313, E133319, E13339, E133391, E133392, E133393, E133399, E13341, E133411, E133412, E133413, E133419, E13349, E133491, E133492, E133493, E133499, E13351, E133511, E133512, E133513, E133519, E133521, E133522, E133523, E133529, E133531, E133532, E133533, E133539, E133541, E133542, E133543, E133549, E133551, E133552, E133553, E133559, E13359, E133591, E133592, E133593, E133599, E1336, E1337X1, E1337X2, E1337X3, E1337X9, E1339, E1340, E1341, E1342, E1343, E1344, E1349, E1351, E1352, E1359, E13610, E13618, E13620, E13621, E13622, E13628, E13630, E13638, E13641, E13649, E1365, E1369, E138, G3289
Endocrine & Nutritional	Failure to thrive (child)	78341	R6251
Endocrine & Nutritional	Feeding difficulties and mismanagement	7833	R633

Endocrine & Nutritional	Lack of expected normal development	78340	R6250
Endocrine & Nutritional	Short stature	78343	R6252
Gastrointestinal	Cancer of colon, stomach, GI organs	151, 152, 153, 156, 158, 159, 2090, 20910, 20911, 20912, 20913, 20914, 20915, 20916, 20923, 2302, 2303, 2307, 2309, V1000, V1004, V1005, V1009	C160, C161, C162, C163, C164, C165, C166, C168, C169, C49A2, C49A3, C7A092, D002, Z85028, C180, C181, C182, C183, C184, C185, C186, C187, C188, C189, C260, C49A4, C7A020, C7A021, C7A022, C7A023, C7A024, C7A025, C7A029, D010, Z85038, C170, C171, C172, C173, C178, C179, C23, C240, C241, C248, C249, C261, C269, C451, C480, C481, C482, C488, C49A0, C49A9, C7A010, C7A011, C7A012, C7A019, D0140, D0149, D017, D019, Z8500, Z85068, Z8507, Z8509
Gastrointestinal	Fecal impaction	56032	K5641
Gastrointestinal	GERD	53081	K21, K210, K219
Gastrointestinal	Intestinal atresia	7511, 7512	Q419, Q429
Gastrointestinal	Intestinal obstruction	560	K56, K560, K561, K562, K563, K564, K5641, K5649, K565, K566, K5660, K5669, K567
Gastrointestinal	Meconium obstruction in fetus or newborn	7771	P760
Gastrointestinal	Meconium peritonitis	7776	P780
Gastrointestinal Symptoms	Abdominal pain	7890	R10, R100, R101, R1010, R1011, R1012, R1013, R102, R103, R1030, R1031, R1032, R1033, R108, R1081, R10811, R10812, R10813, R10814, R10815, R10816, R10817, R10819, R1082, R10821, R10822, R10823, R10824, R10825, R10826, R10827, R10829, R1083, R1084, R109
Gastrointestinal Symptoms	Constipation	5640	K590, K5900, K5901, K5902, K5909
Gastrointestinal Symptoms	Diarrhea	78791	R197
Gastrointestinal Symptoms	Eosinophilic esophagitis	53013	K200

Gastrointestinal Symptoms	Nausea or vomiting	5362, 56987, 78701, 78703, 78704, 78720	R110, R111, R1110, R1111, R1112, R1113, R1114, R112
Genitourinary	Male infertility	606	N46, N460, N4601, N4602, N46021, N46022, N46023, N46024, N46025, N46029, N461, N4611, N4612, N46121, N46122, N46123, N46124, N46125, N46129, N468, N469
Hematologic	Venous thromboembolism	415, 453	I8000, I8010, I80209, I803, I80219, I808, I808, I808, I808, I809
Hepatobiliary	Abnormal liver serum enzyme levels	7905	R748
Hepatobiliary	Ascites	7895	R180, R188
Hepatobiliary	Cholelithiasis	574	K80, K800, K8000, K8001, K801, K8010, K8011, K8012, K8013, K8018, K8019, K802, K8020, K8021, K803, K8030, K8031, K8032, K8033, K8034, K8035, K8036, K8037, K804, K8040, K8041, K8042, K8043, K8044, K8045, K8046, K8047, K805, K8050, K8051, K806, K8060, K8061, K8062, K8063, K8064, K8065, K8066, K8067, K807, K8070, K8071, K808, K8080, K8081
Hepatobiliary	Chronic Hepatitis	57140, 57141, 57149	K73, K730, K731, K732, K738, K739
Hepatobiliary	Cirrhosis	5715, 5716, 5719	K74, K740, K741, K742, K743, K744, K745, K746, K7460, K7469
Hepatobiliary	Jaundice (not of newborn)	7824	R17
Hepatobiliary	Neonatal jaundice	774, 7740, 7741, 7742, 7743, 77430, 77431, 77439, 7744, 7745, 7746, 7747	P57, P570, P578, P579, P58, P580, P581, P582, P583, P584, P5841, P5842, P585, P588, P589, P59, P590, P591, P592, P5920, P5929, P593, P598, P599
Pancreatic	Cyst/pseudocyst of pancreas	5772	K862, K863
Pancreatic	Intestinal Malabsorption	579	K90, K900, K901, K902, K903, K904, K908, K9081, K9089, K909
Pancreatic	Other pancreatic disorders	5772, 5778, 5779	K862, K863, K868, K869

Pancreatic	Pancreatic cancer	1570, 1571, 1572, 1573, 1574, 1578, 1579	C250, C251, C252, C253, C254, C257, C258, C259
Pancreatic	Pancreatic steatorrhea	5794	K903
Pancreatic	Pancreatitis (Acute)	5770	K85, K850, K851, K852, K853, K858, K859
Pancreatic	Pancreatitis (Chronic)	5771	K861
Renal	Dehydration	27651	E860
Renal	Fluid and electrolyte disorders	276, 2766, 27669, 9951	E860, E861, E869, E870, E871, E872, E873, E874, E875, E876, E8770, E8779, E878
Renal	Nephrolithiasis	5920	N200
Respiratory	Asthma	493	J4520, J4521, J4522, J4530, J4531, J4532, J4540, J4541, J4542, J4550, J4551, J4552, J45901, J45902, J45909, J45990, J45991, J45998
Respiratory	Congenital cystic lung	7484	Q330
Respiratory	Nasal Polyposis	471	J33, J330, J331, J338, J339
Respiratory	Respiratory Failure	5185, 5188	J96, J960, J9600, J9601, J9602, J961, J9610, J9611, J9612, J962, J9620, J9621, J9622, J969, J9690, J9691, J9692, J95821, J951, J952, J953, J95822, J80, J9610, J984
Respiratory	Respiratory failure of newborn	77081, 77082, 77083, 77084, 77087, 77088, 77089	P283, P284, P282, P285, P2881, P84, P2889
Respiratory Infection	Aspergillosis associated disease	1173, 4846, 5186	B449, B440, B4481
Respiratory Infection	Nontuberculous mycobacterial Infection	031	A31, A310, A311, A312, A318, A319
Respiratory Infection	Bronchiectasis	494	J47, J470, J471, J479
Respiratory Infection	Bronchitis (acute)	466, 4660, 4661, 46611, 46619	J20, J200, J201, J202, J203, J204, J205, J206, J207, J208, J209, J21, J210, J211, J218, J219
Respiratory Infection	Bronchitis (chronic)	491, 4910, 4911, 4912, 49120, 49121, 49122, 4918, 4919	J410, J411, J449, J441, J440, J418, J42

Respiratory Infection	Congenital pneumonia	7700	P23, P230, P231, P232, P233, P234, P235, P236, P238, P239
Respiratory Infection	Personal history of recurrent pneumonia	V1261	Z8701
Respiratory Infection	Pneumonia	00322, 0203, 0204, 0205, 0212, 0221, 0310, 0391, 0521, 0551, 0730, 0830, 1124, 1140, 1144, 1145, 11505, 11515, 11595, 1304, 1363, 480, 481, 482, 483, 484, 485, 486, 5130, 5171	A0103, A0222, A202, A212, A221, A310, A3701, A3711, A430, A481, B012, B052, B0681, B250, B371, B380, B381, B382, B390, B391, B392, B583, B59, B7781, J120, J121, J122, J123, J1281, J1289, J129, J13, J14, J150, J151, J1520, J15211, J15212, J1529, J153, J154, J155, J156, J157, J158, J159, J160, J168, J17, J180, J181, J188, J189, J851
Respiratory Infection	Pseudomonas infections	0417, 4821	B965, J151
Respiratory Infection	Sinusitis (acute)	4610, 4611, 4612, 4613, 4618	J010, J0100, J0101, J011, J0110, J0111, J012, J0120, J0121, J013, J0130, J0131, J014, J0140, J0141, J018, J0180, J0181
Respiratory Infection	Sinusitis (chronic)	4730, 4731, 4732, 4733, 4738	J320, J321, J322, J323, J324, J328
Respiratory Infection	Upper respiratory infection-unspecified	465, 4650, 4658, 4659	J060, J069, J069

Table S3: Results of the potential misclassification analysis for individual conditions.

P-values correspond to the null hypothesis summarized in Methods S2, namely that the estimated effect can be explained by misclassification. P-values are computed for misclassification rates of 0.00295 and 0.00590. The percent and total number of subjects that would need to be misclassified, in order to obtain the estimates reported in Figure 2, on average, are also reported. Across all conditions, on average, 4,153 CF carriers would need to represent misclassified subjects with CF (median 3,055) in order to obtain estimates similar to ours. Note: only misclassification rates of up to 10,000 carriers (i.e. 50.5% misclassification) were simulated.

Condition	P-Value (misclassification .00295)	P-Value (misclassification .00590)	Required Misclassification Percent	Required Misclassified Cases
Abdominal pain	<0.0001	<0.0001	>50.5	>10,000
Abnormal liver serum enzyme levels	<0.0001	<0.0001	31.22	6,182
Acute Bronchitis	0.0380	0.0452	7.08	1,401
Acute Sinusitis	0.0003	0.0004	44.28	8,769
Ascites	<0.0001	<0.0001	33.86	6,706
Aspergillosis associated disease	0.0671	0.1793	0.99	196
Asthma	<0.0001	<0.0001	24.97	4,945
Bronchiectasis	<0.0001	0.0003	1.72	341
Cachexia and Adult Failure to thrive	0.0049	0.0080	5.91	1,171
Cancer of colon, stomach, GI organs	0.0279	0.0295	41.86	8,290
Cholelithiasis	0.0020	0.0023	23.16	4,587
Chronic Bronchitis	0.0135	0.0235	3.33	660
Chronic Hepatitis	0.0132	0.0142	42.62	8,439
Chronic Sinusitis	<0.0001	<0.0001	8.62	1,708
Cirrhosis	0.3373	0.3840	1.05	208
Congenital cystic lung	0.0707	0.0913	3.39	672
Congenital pneumonia	0.0020	0.0021	>50.5	>10,000
Constipation	<0.0001	<0.0001	26.37	5,222
Cyst/pseudocyst of pancreas	0.0021	0.0027	16.91	3,349
Dehydration	<0.0001	<0.0001	28.22	5,588
Diabetes (Type 1 or Secondary)	<0.0001	<0.0001	25.55	5,060
Diarrhea	<0.0001	<0.0001	35.17	6,964
Eosinophilic Esophagitis	0.0523	0.0540	>50.5	10,000
Failure to thrive (child)	<0.0001	<0.0001	15.43	3,055
Fecal impaction	0.0186	0.0205	21.38	4,234

Feeding difficulties and mismanagement	<0.0001	<0.0001	44.12	8,736
Fluid and electrolyte disorders	<0.0001	<0.0001	17.05	3,375
GERD	<0.0001	<0.0001	12.77	2,529
Hemoptysis	0.0025	0.0064	3.19	631
Hypertrophic osteoarthropathy/clubbing	0.1104	0.1238	6.05	1,198
Intestinal atresia	0.0384	0.0491	5.65	1,118
Intestinal Malabsorption	0.0013	0.0027	4.44	878
Intestinal obstruction	0.0075	0.0124	4.61	913
Jaundice (not of newborn)	<0.0001	<0.0001	>50.5	>10,000
Lack of Expected Normal Development	<0.0001	0.0001	11.32	2,241
Male infertility	<0.0001	<0.0001	>50.5	>10,000
Meconium obstruction in fetus or newborn	0.0033	0.0046	10.5	2,079
Meconium peritonitis	0.0808	0.1025	5.35	1,060
Nasal Polyposis	0.0083	0.0157	3.49	691
Nausea or Vomiting	<0.0001	<0.0001	>50.5	>10,000
Neonatal jaundice	<0.0001	<0.0001	>50.5	>10,000
Nephrolithiasis	0.0038	0.0044	17.26	3,418
Nontuberculous Mycobacterial Infection	0.0396	0.0847	1.77	351
Osteopenia	0.0501	0.0699	3.37	668
Osteoporosis	0.3784	0.4500	0.63	124
Other Pancreatic Disorders	<0.0001	<0.0001	2.34	463
Pancreatic Cancer	0.0212	0.0227	>50.5	>10,000
Pancreatic Steatorrhea	0.1523	0.2623	1.08	213
Pancreatitis (Acute)	<0.0001	<0.0001	36.29	7,185
Pancreatitis (Chronic)	<0.0001	<0.0001	33.91	6,715
Personal history of recurrent pneumonia	0.0002	0.0003	14.48	2,868
Pneumonia	0.0005	0.0014	3.43	679
Pseudomonas Infections	0.3990	0.8433	0.26	52
Respiratory Failure	<0.0001	0.0001	3.47	687
Respiratory Failure of Newborn	<0.0001	<0.0001	49.49	9,801
Scoliosis	0.0032	0.0036	23.83	4,719
Short stature	0.0003	0.0005	13.4	2,654
Upper Respiratory Infection-Unspecified	<0.0001	<0.0001	>50.5	>10,000

Venous thromboembolism	0.0573	0.0675	6.29	1,246

Table S4: Results of potential misclassification across all conditions.

We performed one million simulation trials using both of the misclassification rates described. The number of conditions (out of 59) for which the simulated odds ratio was greater than the results reported in Figure 2 is summarized across the million trials. For the lower misclassification rate, there were never more than 14 conditions for which we could obtain simulated results, and 99% of the trials returned 7 or fewer conditions with similar estimates to those in Figure 2. For the greater misclassification rate, there were never more than 17 conditions for which we could obtain simulated results, and 99% of the trials returned 10 or fewer conditions, with similar estimates to those in Figure 2. These findings suggest the results across all conditions in Figure 2 are almost certainly not attributable to misclassification bias.

	Misclassification Rate = 0.00295		Misclassification Rate = 0.00590			
**Number of Conditions with OR > results in Table 2**	**Total Trials**	**% of Trials**	**Number of Conditions with OR > results in Table 2**	**Total Trials**	**% of Trials**	**Number of Conditions with OR > results in Table 2**
0	106,632	0.1066	0	16,771	0.0168	0.0168
1	268,597	0.2686	1	117,026	0.1170	0.1338
2	302,408	0.3024	2	252,718	0.2527	0.3865
3	199,150	0.1992	3	282,506	0.2825	0.6690
4	87,957	0.088	4	195,232	0.1952	0.8643
5	27,410	0.0274	5	93,169	0.0932	0.9574
6	6,487	0.0065	6	32,112	0.0321	0.9895
7	1,172	0.0012	7	8,396	0.0084	0.9979
8	167	0.0002	8	1,765	0.0018	0.9997
9	17	0.0000	9	265	0.0003	1.0000
10	2	0.0000	10	37	0.0000	1.0000
11	1	0.0000	11	2	0.0000	1.0000
12	0	0.0000	12	1	0.0000	1.0000
>12	0	0.0000	>12	0	0.0000	1.0000
Median	2		Median	3		
Mean	2.01		Mean	2.99		

Table S5: Results of multiple comparison analysis for empirical false discovery rate.

We used a simulation analysis to estimate the potential for false discovery associated with multiple hypothesis testing. For each simulation trial, we drew simulated carrier and matched control cohorts using only non-carrier enrollees. We then computed the number of conditions that resulted in p-values ≤ those obtained in our study. Below the number of conditions that resulted in similar significance levels are reported across simulation trials.

Number of Conditions with p-value ≤ study value	Total Trials	% of Trials	Cumulative Percent
0	6,470	0.647	0.647
1	2,860	0.286	0.933
2	580	0.058	0.991
3	80	0.008	0.999
4	10	0.001	1.000
Median	0		
Mean	0.43		

Table S6: Results of sensitivity analysis for association bias associated with screening.

CF carriers with procedure codes for chloride sweat testing (CPT code 89230) or expanded CF screening panels (CPT codes 81221, 81222, or 81223) were excluded for this sensitivity analysis. A total of 1,276 CF carriers were excluded, based on evidence that CF was suspected prior to genetic screening. By removing CF carriers with more severe disease presentations (i.e., suspected of having CF), results using the excluded cohort are intentionally biased towards the null hypothesis, especially those conditions often attributed to CF. Results using the primary study cohort and the reduced cohort are presented below.

Together, these results suggest that ascertainment bias cannot explain our general findings. For some of the rare conditions that would likely lead to suspicion of CF (e.g., pancreatic steatorrhea, hypertrophic osteoarthropathy/clubbing, meconium peritonitis, or congenital pneumonia) statistical significance fell below a 0.05% threshold with the reduced cohort. However, most results remained significant, and the estimated effects for both cohorts were nearly identical for the vast majority of the conditions analyzed. Moreover, many conditions that are highly characteristic of CF (e.g., aspergillosis, bronchiectasis, recurrent pneumonia, and pseudomonas or nontuberculous mycobacterial infection) remained highly significant.

	**Primary Cohort N=19,802**		**Reduced Cohort (Excluding cases with CF suspicion) N=18,526**	
	**OR (95% CI)**	***p*-value**	**OR (95% CI)**	***p*-value**
Abdominal pain	1.4 (1.35-1.45)	<0.001	1.39 (1.34-1.44)	<0.001
Acute Bronchitis	1.04 (0.99-1.08)	0.087	1.03 (0.99-1.08)	0.159
Chronic Bronchitis	1.24 (1.05-1.47)	0.013	1.16 (0.97-1.39)	0.093
Upper Respiratory Infection-Unspecified	1.2 (1.16-1.25)	<0.001	1.19 (1.15-1.24)	<0.001
Ascites	1.69 (1.39-2.06)	<0.001	1.62 (1.33-1.98)	<0.001
Aspergillosis associated disease	3.89 (1.45-10.44)	0.007	2.78 (0.93-8.29)	0.067
Asthma	1.36 (1.29-1.43)	<0.001	1.34 (1.28-1.41)	<0.001
Bronchiectasis	5.62 (3.85-8.21)	<0.001	4.47 (2.93-6.81)	<0.001
Cancer of colon, stomach, GI organs	1.44 (1.01-2.05)	0.042	1.4 (0.98-2)	0.068
Pancreatic Cancer	3.13 (1.02-9.55)	0.046	3.13 (1.02-9.55)	0.046
Constipation	1.32 (1.24-1.41)	<0.001	1.28 (1.2-1.37)	<0.001
Cholelithiasis	1.14 (1.04-1.25)	0.004	1.14 (1.04-1.25)	0.004
Cirrhosis	1.13 (0.72-1.77)	0.603	1.07 (0.67-1.72)	0.774
Hypertrophic osteoarthropathy/clubbing	3.33 (0.94-11.81)	0.062	1.67 (0.34-8.26)	0.532
Congenital cystic lung	4.38 (1.59-12.06)	0.004	5.83 (1.96-17.36)	0.002
Dehydration	1.39 (1.27-1.51)	<0.001	1.39 (1.27-1.52)	<0.001
Diabetes (Type 1 or Secondary)	1.49 (1.4-1.59)	<0.001	1.47 (1.38-1.57)	<0.001

Diarrhea	1.18 (1.11-1.25)	<0.001	1.16 (1.1-1.23)	<0.001
Eosinophilic Esophagitis	1.42 (0.96-2.1)	0.082	1.48 (1-2.2)	0.051
Cachexia and Adult Failure to thrive	2.69 (1.41-5.16)	0.003	2.4 (1.21-4.78)	0.013
Failure to thrive (child)	2.78 (2.28-3.41)	<0.001	2.84 (2.09-3.87)	<0.001
Fecal impaction	2.3 (1.29-4.08)	0.005	2.42 (1.33-4.41)	0.004
Feeding difficulties and mismanagement	2.41 (2.09-2.78)	<0.001	3.38 (2.81-4.06)	<0.001
Fluid and electrolyte disorders	1.26 (1.19-1.35)	<0.001	1.25 (1.17-1.34)	<0.001
GERD	1.16 (1.11-1.22)	<0.001	1.13 (1.07-1.19)	<0.001
Hemoptysis	1.63 (1.22-2.18)	0.001	1.51 (1.12-2.05)	0.007
Chronic Hepatitis	1.73 (1.12-2.68)	0.014	1.69 (1.07-2.66)	0.023
Male infertility	5.09 (4.27-6.07)	<0.001	4.92 (4.12-5.89)	<0.001
Intestinal atresia	2.94 (1.35-6.43)	0.007	3.5 (1.33-9.19)	0.011
Intestinal Malabsorption	1.3 (1.12-1.51)	0.001	1.21 (1.03-1.42)	0.017
Intestinal obstruction	1.27 (1.07-1.52)	0.007	1.23 (1.02-1.48)	0.03
Jaundice (not of newborn)	1.66 (1.39-1.97)	<0.001	1.79 (1.46-2.2)	<0.001
Abnormal liver serum enzyme levels	1.36 (1.18-1.57)	<0.001	1.29 (1.11-1.49)	0.001
Nontuberculous Mycobacterial Infection	2.75 (1.32-5.74)	0.007	1.84 (0.77-4.38)	0.167
Meconium obstruction in fetus or newborn	13 (4.63-36.46)	<0.001	55 (7.1-426)	<0.001
Meconium peritonitis	7.5 (2.12-26.58)	0.002	20 (2.24-178.94)	0.007
Nasal Polyposis	1.37 (1.1-1.7)	0.004	1.32 (1.05-1.64)	0.016
Nausea or Vomiting	1.32 (1.26-1.39)	<0.001	1.3 (1.24-1.37)	<0.001
Nephrolithiasis	1.14 (1.04-1.25)	0.007	1.14 (1.04-1.26)	0.007
Neonatal jaundice	2.15 (1.97-2.35)	<0.001	2.72 (2.45-3.03)	<0.001
Osteopenia	1.13 (0.98-1.31)	0.091	1.1 (0.95-1.27)	0.21
Osteoporosis	1.05 (0.85-1.3)	0.655	1.03 (0.83-1.28)	0.807
Other Pancreatic Disorders	4.04 (2.93-5.56)	<0.001	3.18 (2.25-4.49)	<0.001
Cyst/pseudocyst of pancreas	2.78 (1.61-4.8)	<0.001	2.22 (1.23-4)	0.008
Pancreatitis (Acute)	2.5 (2.08-3)	<0.001	2.29 (1.89-2.78)	<0.001
Pancreatitis (Chronic)	6.76 (4.87-9.39)	<0.001	5.69 (3.99-8.12)	<0.001
Pancreatic Steatorrhea	10 (2.5-39.98)	0.001	8.33 (1.99-34.87)	0.004
Pneumonia	1.16 (1.07-1.26)	<0.001	1.15 (1.05-1.25)	0.002
Personal history of recurrent pneumonia	2.76 (1.79-4.25)	<0.001	2.17 (1.33-3.54)	0.002
Congenital pneumonia	3.95 (2.01-7.77)	<0.001	5 (1.98-12.6)	0.001
Pseudomonas Infections	2 (1.12-3.57)	0.019	1.89 (1.02-3.5)	0.042
Respiratory Failure	1.36 (1.21-1.54)	<0.001	1.25 (1.09-1.42)	0.001
Respiratory Failure of Newborn	2.22 (1.87-2.63)	<0.001	2.86 (2.29-3.57)	<0.001
Scoliosis	1.24 (1.06-1.44)	0.006	1.25 (1.08-1.46)	0.004

Short stature	2.41 (1.6-3.64)	<0.001	3 (1.87-4.81)	<0.001
Lack of Expected Normal Development	2.06 (1.52-2.8)	<0.001	2.2 (1.47-3.31)	<0.001
Acute Sinusitis	1.09 (1.04-1.15)	0.001	1.09 (1.03-1.15)	0.002
Chronic Sinusitis	1.14 (1.09-1.21)	<0.001	1.13 (1.07-1.19)	<0.001
Venous thromboembolism	1.13 (0.98-1.31)	0.099	1.14 (0.98-1.32)	0.091

Figure S1: Prevalence and natural log (LN) odds ratios for each condition analyzed for subjects with CF and matched cohort. Results reported are similar to those in Figure 2, but for subjects with CF.

Organ System & Condition	Prevalence (per 1,000 enrollees) CF Cohort N = 13,305	Matched Cohort N = 66,525	Odds Ratio (95% CI)	P−Value
**Bone**				
Hypertrophic osteoarthropathy/clubbing	5.30	0.06	176.68 (54.73−570.44)	<0.001
Osteopenia	125.72	16.65	11.04 (9.99−12.20)	<0.001
Osteoporosis	86.13	8.48	14.13 (12.42−16.08)	<0.001
Scoliosis	33.91	12.81	2.75 (2.41−3.14)	<0.001
**Endocrine & Nutritional**				
Cachexia and adult failure to thrive	28.42	0.40	75.58 (48.02−118.97)	<0.001
Dehydration	110.12	21.50	5.67 (5.21−6.18)	<0.001
Diabetes (Type 1 or secondary)	345.95	40.06	14.50 (13.59−15.47)	<0.001
Diabetes (Type 2)	253.95	40.89	9.95 (9.29−10.66)	<0.001
Failure to thrive (child)	104.82	4.91	27.25 (23.58−31.50)	<0.001
Feeding difficulties and mismanagement	62.43	8.54	8.84 (7.76−10.06)	<0.001
Fluid and electrolyte disorders	222.83	40.81	6.89 (6.46−7.36)	<0.001
Lack of expected normal development	19.27	4.12	4.90 (4.02−5.96)	<0.001
Short stature	27.84	4.70	6.33 (5.32−7.54)	<0.001
**Gastrointestinal**				
Cancer of colon, stomach, GI organs	7.42	1.56	4.85 (3.54−6.65)	<0.001
Fecal impaction	11.56	0.62	19.00 (12.86−28.08)	<0.001
GERD	375.53	77.19	7.37 (6.99−7.77)	<0.001
Intestinal atresia	5.68	0.12	50.39 (21.70−116.97)	<0.001
Intestinal obstruction	108.00	4.82	24.74 (21.51−28.47)	<0.001
Meconium obstruction in fetus or newborn	8.77	0.06	358.87 (112.50−1144.78)	<0.001
Meconium peritonitis	2.79	0.02	146.83 (19.99−1078.59)	<0.001
**Gastrointestinal Symptoms**				
Abdominal pain	414.26	191.05	3.04 (2.90−3.18)	<0.001
Constipation	262.24	56.61	6.05 (5.70−6.41)	<0.001
Diarrhea	133.33	61.72	2.37 (2.22−2.54)	<0.001
Eosinophilic esophagitis	4.24	1.29	3.30 (2.25−4.83)	<0.001
Nausea or vomiting	235.16	95.92	2.92 (2.77−3.09)	<0.001
**Genitourinary**				
Male infertility	29.11	3.59	9.01 (7.10−11.44)	<0.001
**Hematologic**				
Anemia	132.56	42.97	3.60 (3.34−3.87)	<0.001
Venous thromboembolism	59.25	5.93	10.75 (9.35−12.37)	<0.001
**Hepatobiliary**				
Abnormal liver serum enzyme levels	42.97	6.74	6.68 (5.79−7.70)	<0.001
Ascites	27.84	3.06	9.34 (7.69−11.36)	<0.001
Cholelithiasis	44.89	11.35	4.24 (3.74−4.80)	<0.001
Chronic hepatitis	5.97	0.42	14.23 (8.74−23.16)	<0.001
Cirrhosis	38.92	1.37	29.72 (23.04−38.34)	<0.001
Jaundice (not of newborn)	12.52	4.62	2.81 (2.26−3.50)	<0.001
Neonatal jaundice	18.59	17.61	1.07 (0.90−1.27)	0.446
**Pancreatic**				
Cyst/pseudocyst of pancreas	11.46	0.31	37.57 (22.28−63.34)	<0.001
Intestinal malabsorption	207.90	5.65	44.84 (39.55−50.84)	<0.001
Other pancreatic disorders	543.55	0.79	1312.58 (948.29−1816.82)	<0.001
Pancreatic steatorrhea	69.65	0.02	3875.58 (545.08−27555.63)	<0.001
Pancreatitis (acute)	50.39	2.43	22.69 (18.58−27.71)	<0.001
Pancreatitis (chronic)	45.47	0.58	85.32 (58.59−124.26)	<0.001
**Renal**				
Nephrolithiasis	65.90	15.07	4.77 (4.29−5.31)	<0.001
**Respiratory**				
Asthma	418.40	105.21	5.92 (5.64−6.21)	<0.001
Congenital cystic lung	8.77	0.12	76.72 (33.57−175.33)	<0.001
Hemoptysis	106.36	1.89	63.73 (51.69−78.57)	<0.001
Nasal polyposis	150.48	2.20	81.82 (67.37−99.37)	<0.001
Respiratory failure	366.28	13.03	44.15 (40.29−48.38)	<0.001
Respiratory failure of newborn	13.87	3.60	4.25 (3.38−5.34)	<0.001
**Respiratory Infection**				
Acute sinusitis	112.72	61.31	1.97 (1.83−2.11)	<0.001
Aspergillosis associated disease	99.52	0.17	627.75 (325.56−1210.43)	<0.001
Bronchiectasis	413.87	0.44	1654.41 (1064.87−2570.34)	<0.001
Chronic ethmoidal sinusitis	117.63	3.55	38.25 (32.62−44.84)	<0.001
Chronic frontal sinusitis	70.42	1.98	38.31 (31.10−47.19)	<0.001
Chronic maxillary sinusitis	178.61	9.46	23.23 (20.92−25.78)	<0.001
Chronic sinusitis, other	195.57	4.70	53.82 (46.84−61.84)	<0.001
Chronic sinusitis, unspecified	457.03	60.58	12.64 (11.98−13.35)	<0.001
Chronic sphenoidal sinusitis	42.29	0.73	60.68 (43.52−84.61)	<0.001
Congenital pneumonia	2.60	0.12	22.50 (9.29−54.50)	<0.001
Nontuberculous mycobacterial infection	69.65	0.15	486.74 (242.36−977.56)	<0.001
Personal history of recurrent pneumonia	19.08	0.98	19.69 (14.46−26.80)	<0.001
Pneumonia	454.24	44.18	16.66 (15.72−17.65)	<0.001
Pseudomonas infections	312.43	0.31	1337.17 (817.55−2187.03)	<0.001

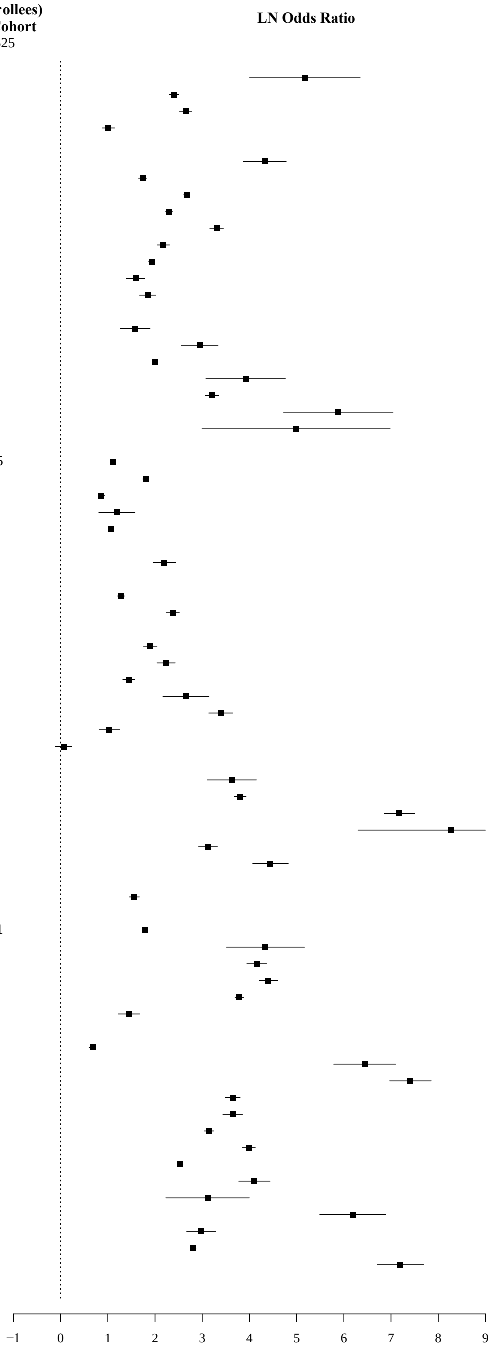

Figure S2: Prevalence and natural log (LN) odds ratios for each CF-related condition in the independent validation cohort.

Organ System & Condition	Prevalence (per 1,000 enrollees) CF Carriers N = 2,185	Prevalence (per 1,000 enrollees) Matched Cohort N = 10,924	Odds Ratio (95% CI)	P−Value
**Bone**				
Hypertrophic osteoarthropathy/clubbing	0.00	0.00		NA
Osteopenia	7.32	10.25	0.70 (0.41−1.20)	0.197
Osteoporosis	5.49	4.03	1.37 (0.72−2.63)	0.335
Scoliosis	9.61	6.96	1.39 (0.85−2.25)	0.188
**Endocrine & Nutritional**				
Cachexia and adult failure to thrive	0.00	0.18		NA
Dehydration	31.58	15.75	2.05 (1.54−2.72)	<0.001
Diabetes (Type 1 or secondary)	63.16	37.26	1.77 (1.45−2.16)	<0.001
Feeding difficulties and mismanagement	5.49	2.29	2.40 (1.21−4.78)	0.013
Fluid and electrolyte disorders	60.41	38.91	1.60 (1.31−1.97)	<0.001
Lack of expected normal development	0.92	0.37	2.50 (0.46−13.65)	0.290
Short stature	0.00	0.00		NA
**Gastrointestinal**				
Cancer of colon, stomach, GI organs	2.75	1.10	2.50 (0.94−6.66)	0.067
Fecal impaction	0.92	0.37	2.52 (0.46−13.85)	0.289
GERD	85.58	72.96	1.19 (1.01−1.41)	0.039
Intestinal atresia	2.29	0.18	12.50 (2.43−64.43)	0.003
Intestinal obstruction	11.90	5.40	2.23 (1.40−3.56)	0.001
**Gastrointestinal Symptoms**				
Abdominal pain	348.28	255.13	1.61 (1.45−1.78)	<0.001
Constipation	51.72	40.28	1.30 (1.05−1.62)	0.015
Diarrhea	73.23	56.21	1.34 (1.11−1.60)	0.002
Eosinophilic esophagitis	1.83	0.46	4.00 (1.07−14.90)	0.039
Nausea or vomiting	105.72	72.41	1.52 (1.30−1.78)	<0.001
**Hematologic**				
Venous thromboembolism	20.59	11.44	1.82 (1.29−2.56)	0.001
**Hepatobiliary**				
Abnormal liver serum enzyme levels	4.58	5.77	0.79 (0.41−1.55)	0.496
Ascites	7.78	3.20	2.43 (1.36−4.33)	0.003
Cholelithiasis	36.61	27.00	1.37 (1.07−1.77)	0.014
Chronic hepatitis	0.92	0.64	1.43 (0.30−6.88)	0.656
Cirrhosis	0.00	1.19		NA
Jaundice (not of newborn)	5.03	2.47	2.04 (1.01−4.12)	0.047
**Pancreatic**				
Cyst/pseudocyst of pancreas	1.37	0.18	7.50 (1.25−44.88)	0.027
Intestinal malabsorption	9.15	6.50	1.41 (0.86−2.32)	0.176
Other pancreatic disorders	1.37	1.10	1.25 (0.35−4.44)	0.729
Pancreatic steatorrhea	0.00	0.00		NA
Pancreatitis (acute)	6.86	3.39	2.04 (1.12−3.73)	0.021
Pancreatitis (chronic)	1.37	0.73	1.88 (0.50−7.07)	0.353
**Renal**				
Nephrolithiasis	28.83	19.77	1.47 (1.11−1.96)	0.008
**Respiratory**				
Asthma	117.62	69.75	1.80 (1.54−2.09)	<0.001
Hemoptysis	3.20	2.20	1.46 (0.63−3.38)	0.380
Nasal polyposis	5.49	2.20	2.50 (1.25−5.00)	0.010
Respiratory failure	13.27	8.70	1.54 (1.01−2.34)	0.045
**Respiratory Infection**				
Acute bronchitis	168.42	146.56	1.19 (1.05−1.35)	0.008
Acute sinusitis	100.23	81.29	1.27 (1.08−1.48)	0.003
Aspergillosis associated disease	0.00	0.09		NA
Bronchiectasis	0.00	0.64		NA
Chronic bronchitis	7.78	5.58	1.40 (0.81−2.40)	0.224
Chronic sinusitis	106.64	89.80	1.22 (1.04−1.42)	0.012
Nontuberculous mycobacterial infection	0.00	0.09		NA
Personal history of recurrent pneumonia	0.92	0.37	2.50 (0.46−13.65)	0.290
Pneumonia	30.66	22.06	1.41 (1.07−1.85)	0.015
Pseudomonas infections	0.46	0.18	2.50 (0.23−27.57)	0.454
Upper respiratory infection−unspecified	269.11	225.10	1.29 (1.16−1.44)	<0.001

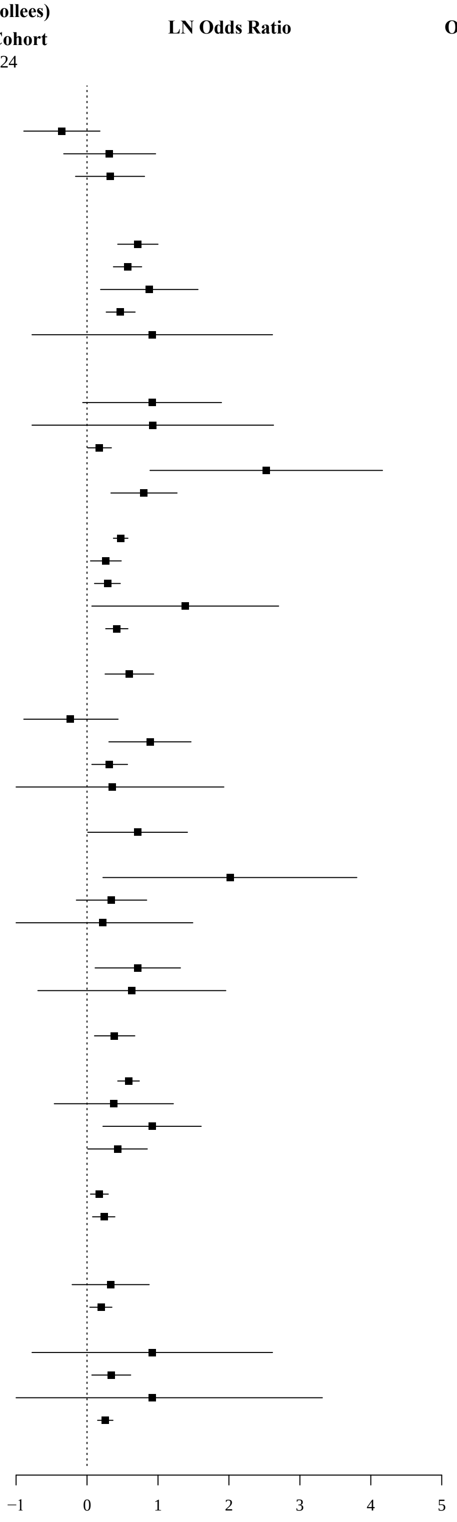

References:

1. Ahmad A, Ahmed A, Patrizio P. Cystic fibrosis and fertility. *Curr Opin Obstet Gynecol.* 2013;25(3):167-172.
2. Assis DN, Debray D. Gallbladder and bile duct disease in Cystic Fibrosis. *J Cystic Fibros.* 2017;16 Suppl 2:S62-s69.
3. Bhagirath AY, Li Y, Somayajula D, Dadashi M, Badr S, Duan K. Cystic fibrosis lung environment and Pseudomonas aeruginosa infection. *BMC Pulm Med.* 2016;16(1):174.
4. Boyle MP. Nonclassic cystic fibrosis and CFTR-related diseases. *Curr Opinion Pulm Med.* 2003;9(6):498-503.
5. Castellani C, Assael BM. Cystic fibrosis: a clinical view. *Cell Mol Life Sci.* 2017;74(1):129-140.
6. Freeman AJ, Ooi CY. Pancreatitis and pancreatic cystosis in Cystic Fibrosis. *J Cystic Fibros.* 2017;16 Suppl 2:S79-s86.
7. Herrmann U, Dockter G, Lammert F. Cystic fibrosis-associated liver disease. *Best Pract Res Clin Gastroenterol.* 2010;24(5):585-592.
8. Knutsen AP, Slavin RG. Allergic bronchopulmonary aspergillosis in asthma and cystic fibrosis. *Clin Dev Immunol.* 2011;2011:843763.
9. Larson JE, Cohen JC. Developmental paradigm for early features of cystic fibrosis. *Pediatr Pulmonol.* 2005;40(5):371-377.
10. Le C, McCrary HC, Chang E. Cystic Fibrosis Sinusitis. *Adv Otoghinolaryngol.* 2016;79:29-37.
11. Naruse S, Kitagawa M, Ishiguro H, Fujiki K, Hayakawa T. Cystic fibrosis and related diseases of the pancreas. *Best Pract Res Clin Gastroenterol.* 2002;16(3):511-526.
12. Nazareth D, Walshaw M. A review of renal disease in cystic fibrosis. *J Cystic Fibros.* 2013;12(4):309-317.
13. Paranjape SM, Zeitlin PL. Atypical cystic fibrosis and CFTR-related diseases. *Clin Rev Allergy Immunol.* 2008;35(3):116-123.
14. Radlovic N. Cystic fibrosis. *Srp Arh Celok Lek.* 2012;140(3-4):244-249.
15. Ratjen F, Doring G. Cystic fibrosis. *Lancet.* 2003;361(9358):681-689.
16. Ronan NJ, Elborn JS, Plant BJ. Current and emerging comorbidities in cystic fibrosis. *Press M.* 2017;46(6 Pt 2):e125-e138.
17. Sathe MN, Freeman AJ. Gastrointestinal, Pancreatic, and Hepatobiliary Manifestations of Cystic Fibrosis. *Pediatr Clin North Am.* 2016;63(4):679-698.
18. Sokol RZ. Infertility in men with cystic fibrosis. *Curr Opinion Pulm Med.* 2001;7(6):421-426.
19. Stalvey MS, Clines GA. Cystic fibrosis-related bone disease: insights into a growing problem. *Curr Opin Endocrinol Diabetes Obes.* 2013;20(6):547-552.
20. Turcios NL. Cystic fibrosis: an overview. *J Clin Gastroenterol.* 2005;39(4):307-317.
21. Classification of cystic fibrosis and related disorders. *J Cystic Fibros.* 2002;1(1):5-8.
22. Abraham JM, Taylor CJ. Cystic Fibrosis & disorders of the large intestine: DIOS, constipation, and colorectal cancer. *J Cystic Fibros.* 2017;16 Suppl 2:S40-s49.
23. Alves Cde A, Lima DS. Cystic fibrosis-related dyslipidemia. *J Bras Pneumol.* 2008;34(10):829-837.
24. Aris RM, Merkel PA, Bachrach LK, et al. Guide to bone health and disease in cystic fibrosis. *J Clin Endocrinol Metab.* 2005;90(3):1888-1896.

25. Baiardini I, Steinhilber G, F DIM, Braido F, Solidoro P. Anxiety and depression in cystic fibrosis. *Minerva Med.* 2015;106(5 Suppl 1):1-8.
26. Balfour-Lynn IM. Asthma in cystic fibrosis. *J R Soc Med.* 2003;96 Suppl 43:30-34.
27. Belkind-Gerson J, Fernandez-Peters A, Furnes R, et al. Pancreatic disorders and cystic fibrosis: Working Group report of the second World Congress of Pediatric Gastroenterology, Hepatology, and Nutrition. *J Pediatr Gastroenterol Nutr.* 2004;39 Suppl 2:S688-694.
28. Bernstein ML, McCusker MM, Grant-Kels JM. Cutaneous manifestations of cystic fibrosis. *Pediatr Dermatol.* 2008;25(2):150-157.
29. Blackman SM, Tangpricha V. Endocrine Disorders in Cystic Fibrosis. *Pediatr Clin North Am.* 2016;63(4):699-708.
30. Borowitz D, Gelfond D. Intestinal complications of cystic fibrosis. *Curr Opinion Pulm Med.* 2013;19(6):676-680.
31. Brennan AL, Geddes DM, Gyi KM, Baker EH. Clinical importance of cystic fibrosis-related diabetes. *J Cystic Fibros.* 2004;3(4):209-222.
32. Bright-Thomas RJ, Webb AK. The heart in cystic fibrosis. *J R Soc Med.* 2002;95 Suppl 41:2-10.
33. Cano Megias M, Gonzalez Albarran O. Cystic fibrosis-related diabetes: a distinct condition. *Endocrinol Nutr.* 2015;62(1):38-44.
34. Chaudry G, Navarro OM, Levine DS, Oudjhane K. Abdominal manifestations of cystic fibrosis in children. *Pediatr Radiol.* 2006;36(3):233-240.
35. Ciofu O, Hansen CR, Hoiby N. Respiratory bacterial infections in cystic fibrosis. *Curr Opinion Pulm Med.* 2013;19(3):251-258.
36. Colombo C. Liver disease in cystic fibrosis. *Curr Opinion Pulm Med.* 2007;13(6):529-536.
37. Constantine S, Au VW, Slavotinek JP. Abdominal manifestations of cystic fibrosis in adults: a review. *Australas Radiol.* 2004;48(4):450-458.
38. Cruz I, Marciel KK, Quittner AL, Schechter MS. Anxiety and depression in cystic fibrosis. *Semin Respir Crit Care Med.* 2009;30(5):569-578.
39. Curran DR, McArdle JR, Talwalkar JS. Diabetes mellitus and bone disease in cystic fibrosis. *Semin Respir Crit Care Med.* 2009;30(5):514-530.
40. Davies JC, Alton EW, Bush A. Cystic fibrosis. *BMJ.*. 2007;335(7632):1255-1259.
41. Debray D, Narkewicz MR, Bodewes F, et al. Cystic Fibrosis-related Liver Disease: Research Challenges and Future Perspectives. *J Pediatr Gastroenterol Nutr.* 2017;65(4):443-448.
42. Diwakar V, Pearson L, Beath S. Liver disease in children with cystic fibrosis. *Paediatr Respir Rev.* 2001;2(4):340-349.
43. Dobson L, Sheldon CD, Hattersley AT. Understanding cystic-fibrosis-related diabetes: best thought of as insulin deficiency? *J R Soc Med.* 2004;97 Suppl 44:26-35.
44. Dodge JA. A millennial view of cystic fibrosis. *Dev Period Med.* 2015;19(1):9-13.
45. Duff AJ. Depression in cystic fibrosis; Implications of The International Depression/Anxiety Epidemiological Study (TIDES) in cystic fibrosis. *Paediatr Respir Rev.* 2015;16 Suppl 1:2-5.
46. Edenborough FP. Women with cystic fibrosis and their potential for reproduction. *Thorax.* 2001;56(8):649-655.

47. Felton IC, Simmonds NJ. Aspergillus and cystic fibrosis: old disease - new classifications. *Curr Opinion Pulm Med.* 2014;20(6):632-638.
48. Flume PA. Pulmonary complications of cystic fibrosis. *Respir Care.* 2009;54(5):618-627.
49. Foweraker J. Recent advances in the microbiology of respiratory tract infection in cystic fibrosis. *Br Med Bull.* 2009;89:93-110.
50. Frayman KB, Kazmerski TM, Sawyer SM. A systematic review of the prevalence and impact of urinary incontinence in cystic fibrosis. *Respriology.* 2018;23(1):46-54.
51. Goralski JL, Lercher DM, Davis SD, Dellon ES. Eosinophilic esophagitis in cystic fibrosis: a case series and review of the literature. *J Cystic Fibros.* 2013;12(1):9-14.
52. Goss CH, Muhlebach MS. Review: Staphylococcus aureus and MRSA in cystic fibrosis. *J Cystic Fibros.* 2011;10(5):298-306.
53. Grasemann H, Ratjen F. Early lung disease in cystic fibrosis. *Lancet Respir Med.* 2013;1(2):148-157.
54. Griffith DE. Emergence of nontuberculous mycobacteria as pathogens in cystic fibrosis. *Am J Respir Crit Care Med.* 2003;167(6):810-812.
55. Haller W, Ledder O, Lewindon PJ, Couper R, Gaskin KJ, Oliver M. Cystic fibrosis: An update for clinicians. Part 1: Nutrition and gastrointestinal complications. *J Gastroenterol Hepatol.* 2014;29(7):1344-1355.
56. Hameed S, Jaffe A, Verge CF. Cystic fibrosis related diabetes (CFRD)--the end stage of progressive insulin deficiency. *Pediatr Pulmonol.* 2011;46(8):747-760.
57. Hamilos DL. Chronic Rhinosinusitis in Patients with Cystic Fibrosis. *J Allergy Clin Immunol Pract.* 2016;4(4):605-612.
58. Hanley-Lopez J, Clement LT. Allergic bronchopulmonary aspergillosis in cystic fibrosis. *Curr Opinion Pulm Med.* 2000;6(6):540-544.
59. Hill UG, Floto RA, Haworth CS. Non-tuberculous mycobacteria in cystic fibrosis. *J R Soc Med.* 2012;105 Suppl 2:S14-18.
60. Kelly T, Buxbaum J. Gastrointestinal Manifestations of Cystic Fibrosis. *Dig Dis Sci.* 2015;60(7):1903-1913.
61. Kent BD, Lane SJ, van Beek EJ, Dodd JD, Costello RW, Tiddens HA. Asthma and cystic fibrosis: a tangled web. *Pediatr Pulmonol.* 2014;49(3):205-213.
62. Kim RJ. Cystic Fibrosis-Related Diabetes in Children: An Update. *Pediatr Ann.* 2016;45(9):e321-326.
63. Kumar N, Balachandran S, Millner PA, Littlewood JM, Conway SP, Dickson RA. Scoliosis in cystic fibrosis: is it idiopathic? *Spine.* 2004;29(18):1990-1995.
64. Labombarda F, Saloux E, Brouard J, Bergot E, Milliez P. Heart involvement in cystic fibrosis: A specific cystic fibrosis-related myocardial changes? *Respir Med.* 2016;118:31-38.
65. Lavelle LP, McEvoy SH, Ni Mhurchu E, et al. Cystic Fibrosis below the Diaphragm: Abdominal Findings in Adult Patients. *Radiographics.* 2015;35(3):680-695.
66. Ledder O, Haller W, Couper RT, Lewindon P, Oliver M. Cystic fibrosis: an update for clinicians. Part 2: hepatobiliary and pancreatic manifestations. *J Gastroenterol Hepatol.* 2014;29(12):1954-1962.
67. Lee AL, Rawlings S, Bennett KA, Armstrong D. Pain and its clinical associations in individuals with cystic fibrosis: A systematic review. *Chron Respir Dis.* 2016;13(2):102-117.

68. Leeuwen L, Fitzgerald DA, Gaskin KJ. Liver disease in cystic fibrosis. *Paediatr Respir Rev.* 2014;15(1):69-74.
69. Leung DH, Narkewicz MR. Cystic Fibrosis-related cirrhosis. *J Cystic Fibros.* 2017;16 Suppl 2:S50-s61.
70. Levy MB. Allergic bronchopulmonary aspergillosis and cystic fibrosis. *Front Biosci.* 2003;8:s579-583.
71. Li D, Wang B, Wang H, Liu Q. Prognostic significance of pulmonary hypertension in patients with cystic fibrosis: A systematic review and meta-analysis. *Medicine.* 2018;97(7):e9708.
72. Liong SY, Awad D, Jones AM, Sukumar SA. The adult cystic fibrosis patient with abdominal pain: what the radiologist needs to know. *Clin Radiol.* 2011;66(2):132-139.
73. Loebinger MR, Bilton D, Wilson R. Upper airway 2: Bronchiectasis, cystic fibrosis and sinusitis. *Thorax.* 2009;64(12):1096-1101.
74. Lord RW, Jones AM, Webb AK, Barry PJ. Pneumothorax in cystic fibrosis: beyond the guidelines. *Paediatr Respir Rev.* 2016;20 Suppl:30-33.
75. Lu KD, Engmann C, Moya F, Muhlebach M. Cystic fibrosis in premature infants. *J Perinatol.* 2011;31(7):504-506.
76. Lusman S, Sullivan J. Nutrition and Growth in Cystic Fibrosis. *Pediatr Clin North Am.* 2016;63(4):661-678.
77. Lusman SS, Grand R. Approach to chronic abdominal pain in Cystic Fibrosis. *J Cystic Fibros.* 2017;16 Suppl 2:S24-s31.
78. Mak GK, Henig NR. Sinus disease in cystic fibrosis. *Clin Rev Allergy Immunol.* 2001;21(1):51-63.
79. Martiniano SL, Davidson RM, Nick JA. Nontuberculous mycobacteria in cystic fibrosis: Updates and the path forward. *Pediatr Pulmonol.* 2017;52(S48):S29-s36.
80. Martiniano SL, Nick JA. Nontuberculous mycobacterial infections in cystic fibrosis. *J Clin Chest Med.* 2015;36(1):101-115.
81. Martiniano SL, Nick JA, Daley CL. Nontuberculous Mycobacterial Infections in Cystic Fibrosis. *J Clin Chest Med.* 2016;37(1):83-96.
82. Masson A, Kirszenbaum M, Sermet-Gaudelus I. Pain is an underestimated symptom in cystic fibrosis. *Curr Opinion Pulm Med.* 2017;23(6):570-573.
83. Minasian C, McCullagh A, Bush A. Cystic fibrosis in neonates and infants. *Early Hum Dev.* 2005;81(12):997-1004.
84. Moheet A, Moran A. CF-related diabetes: Containing the metabolic miscreant of cystic fibrosis. *Pediatr Pulmonol.* 2017;52(S48):S37-s43.
85. Monroe EJ, Pierce DB, Ingraham CR, Johnson GE, Shivaram GM, Valji K. An Interventionalist's Guide to Hemoptysis in Cystic Fibrosis. *Radiographics.* 2018;38(2):624-641.
86. Nankivell G, Caldwell P, Follett J. Urinary incontinence in adolescent females with cystic fibrosis. *Paediatr Respir Rev.* 2010;11(2):95-99.
87. Nash EF, Ohri CM, Stephenson AL, Durie PR. Abdominal pain in adults with cystic fibrosis. *Eur J Gastroenterol Hepatol.* 2014;26(2):129-136.
88. Nathan BM, Laguna T, Moran A. Recent trends in cystic fibrosis-related diabetes. *Curr Opin Endocrinol Diabetes Obes.* 2010;17(4):335-341.
89. Ng MY, Flight W, Smith E. Pulmonary complications of cystic fibrosis. *Clin Radiol.* 2014;69(3):e153-162.

90. Nick JA, Rodman DM. Manifestations of cystic fibrosis diagnosed in adulthood. *Curr Opinion Pulm Med.* 2005;11(6):513-518.
91. Ode KL, Moran A. New insights into cystic fibrosis-related diabetes in children. *Lancet Diabest Endocrinol.* 2013;1(1):52-58.
92. O'Riordan SM, Dattani MT, Hindmarsh PC. Cystic fibrosis-related diabetes in childhood. *Horm Res Paediatr.* 2010;73(1):15-24.
93. O'Shea D, O'Connell J. Cystic fibrosis related diabetes. *Curr Diab Rep.* 2014;14(8):511.
94. O'Sullivan BP, Freedman SD. Cystic fibrosis. *Lancet.* 2009;373(9678):1891-1904.
95. Paccou J, Zeboulon N, Combescure C, Gossec L, Cortet B. The prevalence of osteoporosis, osteopenia, and fractures among adults with cystic fibrosis: a systematic literature review with meta-analysis. *Calci Tissue Int.* 2010;86(1):1-7.
96. Patel L, Dixon M, David TJ. Growth and growth charts in cystic fibrosis. *J R Soc Med.* 2003;96 Suppl 43:35-41.
97. Quittner AL, Barker DH, Snell C, Grimley ME, Marciel K, Cruz I. Prevalence and impact of depression in cystic fibrosis. *Curr Opinion Pulm Med.* 2008;14(6):582-588.
98. Robertson JM, Friedman EM, Rubin BK. Nasal and sinus disease in cystic fibrosis. *Paediatr Respir Rev.* 2008;9(3):213-219.
99. Robertson MB, Choe KA, Joseph PM. Review of the abdominal manifestations of cystic fibrosis in the adult patient. *Radiographics.* 2006;26(3):679-690.
100. Robinson NB, DiMango E. Prevalence of gastroesophageal reflux in cystic fibrosis and implications for lung disease. *Ann Am Thrac Soc.* 2014;11(6):964-968.
101. Salvatore D, Buzzetti R, Baldo E, et al. An overview of international literature from cystic fibrosis registries. Part 4: update 2011. *J Cystic Fibros.* 2012;11(6):480-493.
102. Salvatore D, Buzzetti R, Mastella G. Update of literature from cystic fibrosis registries 2012-2015. Part 6: Epidemiology, nutrition and complications. *Pediatr Pulmonol.* 2017;52(3):390-398.
103. Sathe M, Houwen R. Meconium ileus in Cystic Fibrosis. *J Cystic Fibros.* 2017;16 Suppl 2:S32-s39.
104. Schram CA. Atypical cystic fibrosis: identification in the primary care setting. *Can Fam Physician*2012;58(12):1341-1345, e1699-1704.
105. Scurati-Manzoni E, Fossali EF, Agostoni C, et al. Electrolyte abnormalities in cystic fibrosis: systematic review of the literature. *Pediatr Nephrol.* 2014;29(6):1015-1023.
106. Seitz CS, Gaigl Z, Brocker EB, Trautmann A. Painful wrinkles in the bathtub: association with hyperhidrosis and cystic fibrosis. *Dermatology.* 2008;216(3):222-226.
107. Sinaasappel M, Stern M, Littlewood J, et al. Nutrition in patients with cystic fibrosis: a European Consensus. *J Cystic Fibros.* 2002;1(2):51-75.
108. Singh VK, Schwarzenberg SJ. Pancreatic insufficiency in Cystic Fibrosis. *J Cystic Fibros.* 2017;16 Suppl 2:S70-s78.
109. Soulsby N, Greville H, Coulthard K, Doecke C. Renal dysfunction in cystic fibrosis: is there cause for concern? *Pediatr Pulmonol.* 2009;44(10):947-953.
110. Sparks AA, McGee SJ, Boone CE, Neuringer IP, Jones SK, Aris RM. 'Old' bones in young bodies: the tale of cystic fibrosis. *Curr Opin Endocrinol Diabetes Obes.* 2009;16(6):407-414.
111. Spoonhower KA, Davis PB. Epidemiology of Cystic Fibrosis. *J Clin Chest Med.* 2016;37(1):1-8.

112. Staufer K, Halilbasic E, Trauner M, Kazemi-Shirazi L. Cystic fibrosis related liver disease--another black box in hepatology. *Int J Mol Sc.* 2014;15(8):13529-13549.
113. Stenbit A, Flume PA. Pulmonary complications in adult patients with cystic fibrosis. *The Am J Med Sci.* 2008;335(1):55-59.
114. Strausbaugh SD, Davis PB. Cystic fibrosis: a review of epidemiology and pathobiology. *J Clin Chest Med.* 2007;28(2):279-288.
115. Sueblinvong V, Whittaker LA. Fertility and pregnancy: common concerns of the aging cystic fibrosis population. *J Clin Chest Med.* 2007;28(2):433-443.
116. Takemoto CM. Venous thromboembolism in cystic fibrosis. *Pediatr Pulmonol.* 2012;47(2):105-112.
117. Tombazzi CR, Riely CA. Liver disease in cystic fibrosis. *Rev Med Chil.* 2001;129(9):1071-1078.
118. van Ewijk BE, van der Zalm MM, Wolfs TF, van der Ent CK. Viral respiratory infections in cystic fibrosis. *J Cystic Fibros.* 2005;4 Suppl 2:31-36.
119. Wat D. Impact of respiratory viral infections on cystic fibrosis. *Postgrad Medical J.* 2003;79(930):201-203.
120. Wat D, Doull I. Respiratory virus infections in cystic fibrosis. *Paediatr Respir Rev.* 2003;4(3):172-177.
121. Zirbes J, Milla CE. Cystic fibrosis related diabetes. *Paediatr Respir Rev.* 2009;10(3):118-123; quiz 123.
122. Maisonneuve P, Marshall BC, Lowenfels AB. Risk of pancreatic cancer in patients with cystic fibrosis. *Gut.* 2007;56(9):1327-1328.
123. Bobadilla JL, Macek M, Jr., Fine JP, Farrell PM. Cystic fibrosis: a worldwide analysis of CFTR mutations--correlation with incidence data and application to screening. *Hum Mutat.* 2002;19(6):575-606.
124. Watson MS, Cutting GR, Desnick RJ, et al. Cystic fibrosis population carrier screening: 2004 revision of American College of Medical Genetics mutation panel. *Genet Med.* 2004;6(5):387-391.
125. Cystic Fibrosis Foundation. Carrier Testing for Cystic Fibrosis. 2018; https://www.cff.org/What-is-CF/Testing/Carrier-Testing-for-Cystic-Fibrosis/. Accessed December 11, 2018.
126. Healthcare Cost and Utilization Project. Clinical Classifications Software (CCS) for ICD-9-CM. 2017; https://www.hcup-us.ahrq.gov/toolssoftware/ccs/ccs.jsp. Accessed December 10, 2018.
127. Healthcare Cost and Utilization Project. Beta Clinical Classifications Software (CCS) for ICD-10-CM/PCS. 2018; https://www.hcup-us.ahrq.gov/toolssoftware/ccs10/ccs10.jsp. Accessed December 10, 2018.
